# Data-driven assessment, contextualisation and implementation of 134 variables in the risk for type 2 diabetes: an analysis of Lifelines, a prospective cohort study in the Netherlands

**DOI:** 10.1007/s00125-021-05419-1

**Published:** 2021-03-12

**Authors:** Thomas P. van der Meer, Bruce H. R. Wolffenbuttel, Chirag J. Patel

**Affiliations:** 1 Department of Biomedical Informatics, Harvard Medical School, Boston, MA USA; 2 Department of Endocrinology, University Medical Center Groningen, University of Groningen, Groningen, the Netherlands

**Keywords:** Contextualisation, Data-driven, Identification, Lasso regression, Machine learning, Prediction models, Prospective, Risk variable-wide association study, Type 2 diabetes

## Abstract

**Aims/hypothesis:**

We aimed to assess and contextualise 134 potential risk variables for the development of type 2 diabetes and to determine their applicability in risk prediction.

**Methods:**

A total of 96,534 people without baseline diabetes (372,007 person-years) from the Dutch Lifelines cohort were included. We used a risk variable-wide association study (RV-WAS) design to independently screen and replicate risk variables for 5-year incidence of type 2 diabetes. For identified variables, we contextualised HRs, calculated correlations and assessed their robustness and unique contribution in different clinical contexts using bootstrapped and cross-validated lasso regression models. We evaluated the change in risk, or ‘HR trajectory’, when sequentially assigning variables to a model.

**Results:**

We identified 63 risk variables, with novel associations for quality-of-life indicators and non-cardiovascular medications (i.e., proton-pump inhibitors, anti-asthmatics). For continuous variables, the increase of 1 SD of HbA_1c_, i.e., 3.39 mmol/mol (0.31%), was equivalent in risk to an increase of 0.53 mmol/l of glucose, 19.8 cm of waist circumference, 8.34 kg/m^2^ of BMI, 0.67 mmol/l of HDL-cholesterol, and 0.14 mmol/l of uric acid. Other variables required an increase of >3 SD, which is not physiologically realistic or a rare occurrence in the population. Though moderately correlated, the inclusion of four variables satiated prediction models. Invasive variables, except for glucose and HbA_1c_, contributed little compared with non-invasive variables. Glucose, HbA_1c_ and family history of diabetes explained a unique part of disease risk. Adding risk variables to a satiated model can impact the HRs of variables already in the model.

**Conclusions:**

Many variables show weak or inconsistent associations with the development of type 2 diabetes, and only a handful can reliably explain disease risk. Newly discovered risk variables will yield little over established factors, and existing prediction models can be simplified. A systematic, data-driven approach to identify risk variables for the prediction of type 2 diabetes is necessary for the practice of precision medicine.

**Graphical abstract:**

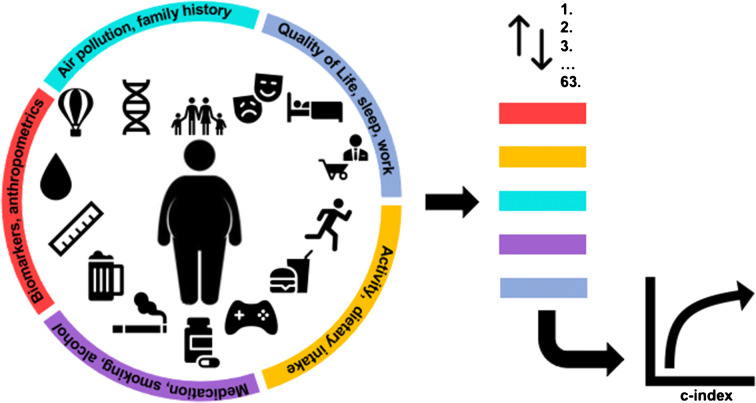

**Supplementary Information:**

The online version contains peer-reviewed but unedited supplementary material available at 10.1007/s00125-021-05419-1.

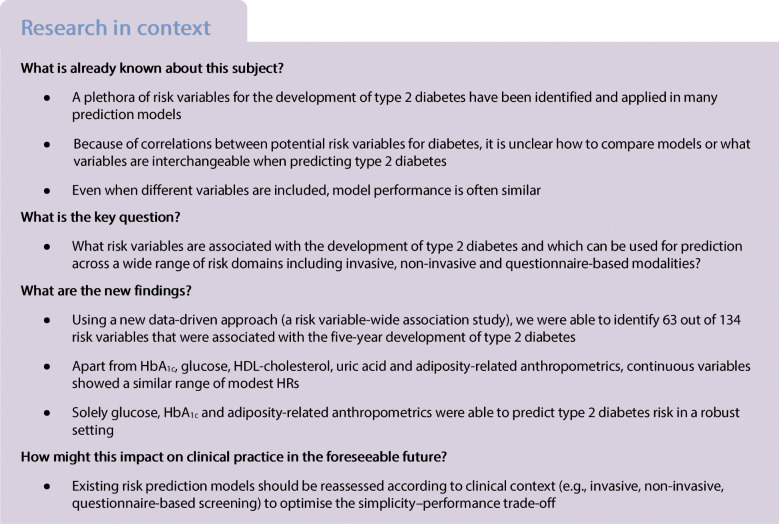



## Introduction

The development of complex, multifactorial diseases such as type 2 diabetes remains poorly understood to date. Though many different risk variables have been identified [1], conventional risk identification approaches often focus on a small set of variables at a time with varying relevant time windows [2]. This leads to fragmentation of the evidence, large inter-study heterogeneity and false positive findings due to multiple testing. Further, the narrow focus makes it hard to contextualise identified risk variables with others.

For some risk variables, such as fasting glucose, specific trajectories are well documented [3]. However, it is unclear how a more diverse set of risk variables may contribute to this heterogeneous rise in glucose. As these variables are often correlated (e.g., adiposity-related traits, BP and lipids), their independent contribution to disease risk with respect to each other is unknown and impossible to dissect in meta-analyses where individual-level data are not available. This lack of insight has led to the development of many risk prediction models, with a recent systematic review identifying 145 different prospective models and scores for the development of type 2 diabetes [2]. Although these models contain different variables, their performance has been shown to be roughly similar [4], suggesting that many variables predict a similar part of disease risk and are thus interchangeable.

Large convenience (e.g., biobanks) and non-convenience cohorts have amassed hundreds to millions of potential risk variables, such as phenotypes and environmental exposures, and it is challenging to identify which variables are predictive of disease outcomes. Data-driven methodologies have been applied to these cohorts to systematically screen and replicate associations between many environmental and nutritional variables and complex diseases [5, 6], to tentatively identify potential risk variables with the strongest statistical support, including larger association sizes and robust inferential statistics such as lower false discovery rates [7].

So far, despite advances in understanding the transition from prediabetes to diabetes, there is no consensus on which risk variables drive the type 2 diabetes epidemic let alone which variables can be used to screen populations who might be at risk. Here, we used a data-driven risk variable-wide association study (RV-WAS) approach to assess associations between 134 known and novel risk variables and the 5-year development of type 2 diabetes. Further, we contextualised the identified variables with each other and investigated their applicability to predict risk in different clinical contexts, including invasive, non-invasive and questionnaire-only variables.

## Methods

### Study population

**Fig. 1:**
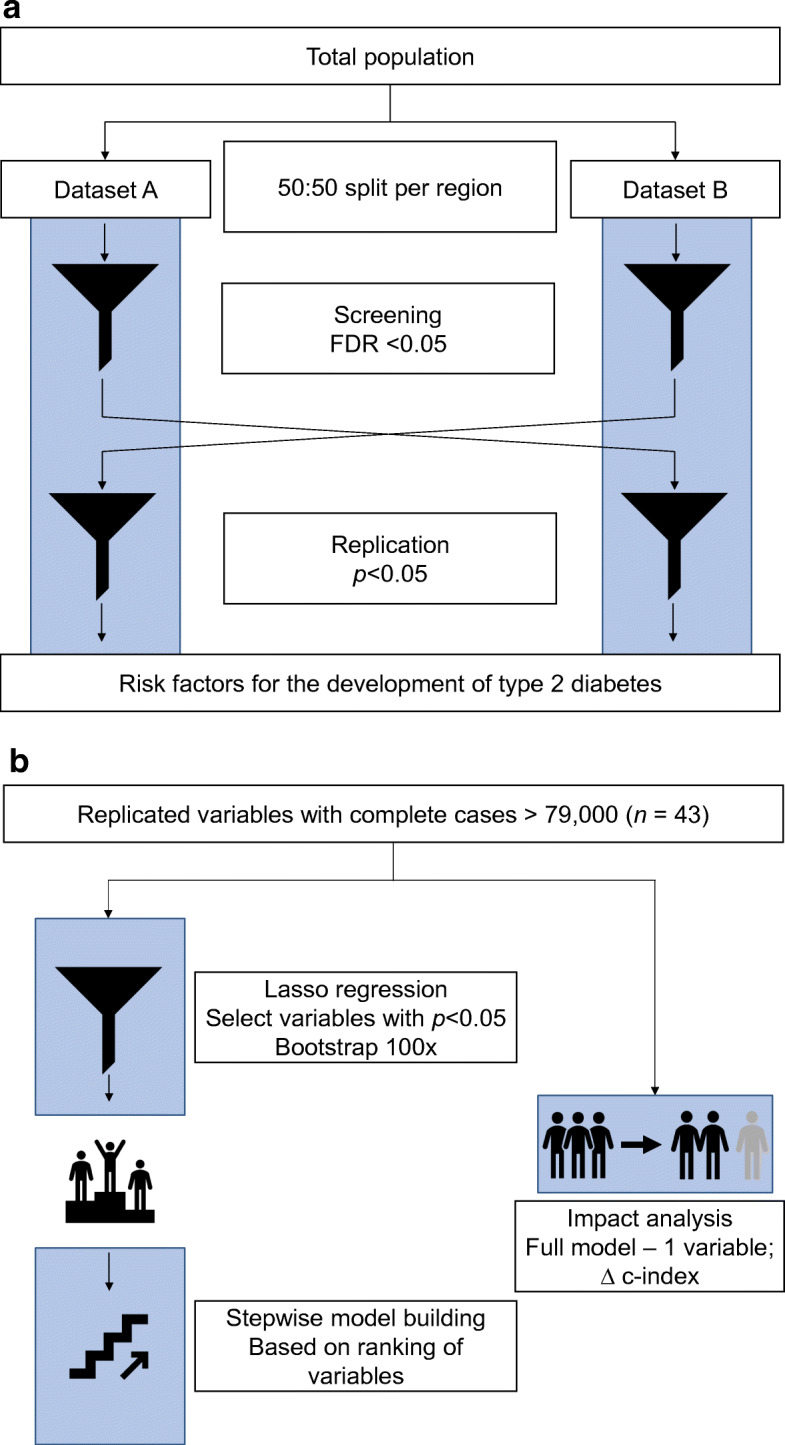
Analytical pipelines to assess risk variables for the development of type 2 diabetes. (**a**) The total population (*n* = 96,534) was split 50:50 into two datasets and 134 variables were screened for associations with the development of type 2 diabetes. Variables with a Benjamini–Yekutieli FDR < 0.05 were crossed over to the other dataset and validated using a *p* value of <0.05. (**b**) Bootstrapped and cross-validated lasso-regression models were used to score robustness of risk variables. Unique risk explained by variables were investigated by recalculating the model discrimination after subtracting a respective variable from the full model. This process was applied in three clinically relevant models (i.e., model including all variables, non-invasive model, questionnaire model)

Lifelines is a multidisciplinary prospective population-based cohort study examining in a three-generation design the health and health-related behaviours of 167,729 people living in the north of the Netherlands. It employs a broad range of procedures to assess the biomedical, socio-demographic, behavioural, physical and psychological factors that contribute to the health and disease of the general population, with a special focus on multi-morbidity and genetics, and has follow-up consisting of questionnaires at median intervals of 1.5 and 3 years, and repeated biochemical measurements after 5 years. We determined diabetes status based on self-reported prior diagnosis, use of diabetes medication, elevated fasting glucose levels ≥7.0 mmol/l, or HbA_1c_ levels of ≥47.5 mmol/mol (6.5%). We excluded all individuals with diabetes at baseline, or without available data at follow-up. Total person-years of follow-up were 372,007. Participant selection is shown in the electronic supplementary material (ESM) Fig. 1. The Lifelines Cohort Study is conducted in accordance with the Declaration of Helsinki and the research code of the University Medical Center Groningen (UMCG). Before study entrance, participants gave informed consent. The study was approved by the UMCG medical ethics review committee.

### Potential risk variables

We included 134 potential risk variables (ESM Table 1). The collection of these variables has been described in detail elsewhere [8]. We chose these 134 variables as they are currently ascertained in the clinic and in epidemiological studies of chronic disease (e.g., Framingham Heart Study) and they are included in a broad array of invasive, non-invasive and self-reported questionnaires on the bulk of the population. In short, measurements were performed by a trained research nurse, electrocardiograms (ECGs) were assessed by a cardiologist, and biochemical analyses were performed in blood and urine. Questionnaires were used to evaluate age, sex, ethnicity, socioeconomic status, smoking status, family history, medication prescription, physical activity and intake of nutrients and vitamins. Sleep quality was assessed using the Epworth Sleepiness Scale, Pittsburgh Sleep Quality Index and the Munich Chronotype Questionnaire, and health-related quality of life was assessed using the RAND 36-Item Health Survey. Further, data on air pollution and noise exposure were available [8]. We included prescription medications that were being used in more than 1% of the study population. To compare risk variables, individual observations for all continuous variables were transformed into z-scores. Variables with less than 20 unique outcomes were treated as categorical.

### Data-driven procedure to identify variables associated with type 2 diabetes risk

The analytical procedure is summarised in Fig. 1a. We created two datasets (A and B) by a 50:50 split based on the first two numbers of the zip code [9]. This way, we aimed to create a geographically independent replication cohort to potentially mitigate healthcare system biases that occur in one region and not another [10]. Each region included both urban and rural areas. We used these datasets to systematically investigate associations between potential risk variables and the development of type 2 diabetes using Cox regression models, adjusting for age and sex. First, we screened individual variables by testing associations between the variable and the development of type 2 diabetes in one dataset. We selected associations that attained a Benjamini–Yekutieli false discovery rate (FDR) <0.05 [7]. Next, we replicated the selected variables in the other dataset, using a threshold of *p* < 0.05. During the analytical procedure, multilevel categorical variables were dichotomised into dummy variables. For replicated risk variables, we recalculated HRs and *p* values in the full population. To improve interpretability for the dichotomised variables that were replicated, we reran the analysis using the original factors in which the most favourable outcome was set as reference.

To analyse sensitivity, we recalculated the HRs of the identified risk variables after excluding individuals with impaired fasting glucose (IFG). We used the more stringent WHO IFG criterion of fasting glucose >6.0 mmol/l [11] to identify individuals with highest baseline glucose levels. When we used the criteria from the ADA (i.e. fasting glucose >5.7 mmol/l), we attained similar results (ESM Fig. 2). Also, we recalculated HRs of the replicated variables while additionally adjusting for IFG. We reported variables that lost nominal significance (*p* ≥ 0.05), or whose HR changed more than 10%.

Next, we aimed to contextualise the replicated risk variables with respect to others. For continuous variables, we calculated the number of SDs needed to achieve the same increase in hazard that 1 SD gives in the variable with the highest HR for the corresponding groups and for all variables. Using these SDs, we recalculated the HRs setting the variable with the highest HR as a reference. Calculations with other reference variables were summarised online in a Shiny application [12].

### Correlation and independence of risk variables

We assessed correlations using Pearson product-moment correlations between numeric variables, polyserial correlations between numeric and ordinal variables, and polychoric correlations between ordinal variables. Correlations were arranged using Ward’s hierarchical clustering algorithm and visualised using a heatmap [13]. We performed the analyses separately for men and women and age tertiles (range: 18–39, 40–48, 49–91 years). Successively, we calculated the effective number of variables for each group taking correlation into account [14].

### Implementation of risk variables in models for different clinical contexts

To determine which variables predict disease risk, we assigned a score to each variable by (1) using 10-fold cross-validated lasso regression to select the optimal model as a function of the tentatively replicated variables [15], (2) assigning one point to the variables that were retained and nominally significant (*p* < 0.05) and (3) bootstrapping the previous steps 100 times. Next, we used Cox proportional hazard models to predict diabetes and assessed the saturation of the model by monitoring the discrimination using the concordance (c-index) while stepwise adding risk variables to the model starting with the highest scoring variable (Fig. 1b). Further, we investigated the unique impact of individual risk variables by removing the respective variable from the model containing all variables of the respective group, after which the difference in discrimination was calculated. We reported changes in the c-index of at least 1% of the original c-index.

We applied the methodology described above to three clinically relevant models. The full model considered all replicated risk variables, the non-invasive model excluded variables that require laboratory measurements or a trained research nurse (i.e., biochemicals, ECG), and the third model solely considered questionnaire-based variables. As lasso regression requires complete data from each individual, we aimed to maximise power while also including as many replicated variables as possible. This resulted in the inclusion of 43 risk variables with available data from more than 79,000 individuals (ESM Table 1). The inclusion of one more variable would have reduced the sample size to 46,743 individuals. For family history, only the aggregated risk factor for first degree relatives was used. To investigate whether differences in discrimination between the investigated models were solely driven by specific variables with the highest scores (e.g., blood/plasma glucose variables, adiposity-related variables), we repeated the analysis after excluding the respective variables.

We performed all analyses using R project software (version: 3.5.2) [16]. The scripts used for the analyses have been summarised and are available in the LIFEWAS package [17].

## Results

### Contextualisation of risk variables for developing type 2 diabetes

**Fig. 2:**
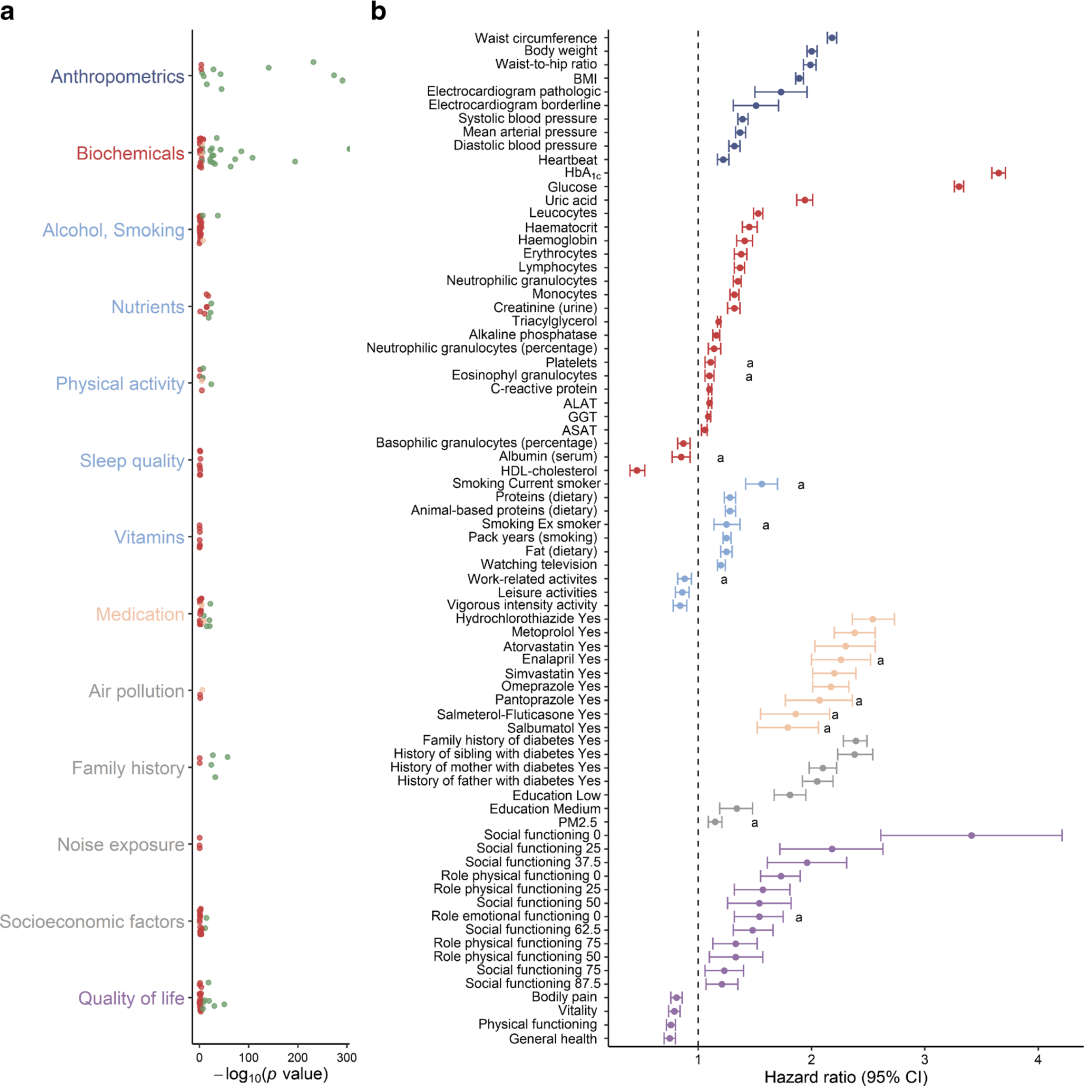
Identified risk variables for the development of type 2 diabetes and their effect estimate. (**a**) Each dot represents one of the 134 risk variables investigated. Green dots (*n* = 53; 40%) were replicated in both pipelines, orange dots (*n* = 10; 7%) were replicated in one pipeline and red dots (*n* = 71; 53%) were not replicated. *p* values were calculated using the complete study population. (**b**) Each dot represents the HR and 95% CI of a variable. Variables with a protective association are shown to the left of the dotted line and variables with a hazardous association are shown on the right-hand side of the dotted line. Colours correspond to the grouped variables in the Manhattan-like plot in (**a**) (dark blue, anthropometrics; red, biochemicals; light blue, lifestyle; orange, medication; grey, predetermined; purple, quality of life). ^a^Variables replicated in one direction. ALAT, alanine aminotransferase; ASAT, aspartate aminotransferase; GGT, γ-glutamytransferase; PM2.5, atmospheric particulate matter with a diameter <2.5 μm

In total, 96,534 participants were included in the study. Study population characteristics are reported in ESM Table 1. In short, the population consisted of slightly fewer men than women (41%) and had a mean age of 45.2 years. A total of 1494 individuals developed type 2 diabetes. Of the 134 variables, we identified 53 variables (40%) in both directions (i.e., screened and replicated in both dataset A to B and B to A), and ten variables (7%) in a single direction (Fig. 2). The *p* values, number of individuals with complete data and the replication in one or two directions are documented in ESM Table 2 and ESM Fig. 3.

We identified categorical risk variables, including a borderline or pathological vs normal ECG (HR: 1.37 and 1.40), being a current (HR: 1.62) or ex-smoker (HR: 1.11) vs non-smoker, and having a prescription for hydrochlorothiazide, metoprolol, atorvastatin, enalapril, simvastatin, omeprazole, pantoprazole, salmeterol-fluticasone, or salbutamol (HR: 2.46 to 1.77). Further, low or medium vs high education (HR: 1.87 and 1.27), having a family history of diabetes (HR: 1.81 for mother, 2.28 for sibling), and several health-related quality-of-life variables were associated with a higher diabetes risk.

**Fig. 3:**
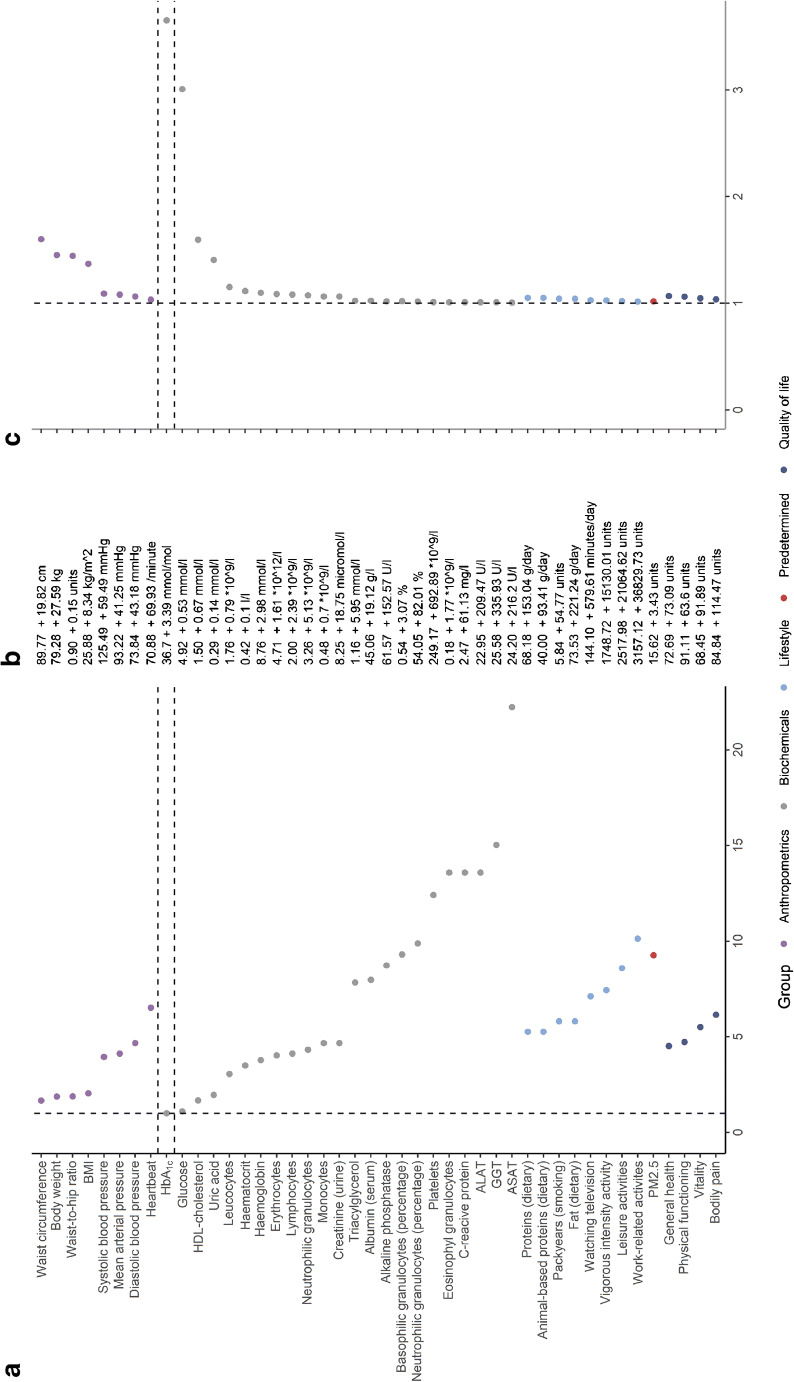
Contextualisation of identified continuous risk variables. (**a**) Each dot represents the number of SDs needed to attain the same hazard as 1 SD increase of the largest risk variables (HbA_1c_). To approximate a hazard similar to a rise in HbA_1c_ from the mean to the diabetes threshold, SDs should be multiplied by a factor of 3. (**b**) The population mean of each variable and the required increase in the respective variable to increase the hazard for developing type 2 diabetes as much as 1 SD of HbA_1c_. Other risk variables can be set as reference via the application online [12]. (**c**) Each dot represents the HR of a risk variable after correction for the hazard of HbA_1c_. To compare variables with a protective and hazardous effect, absolute coefficients were used. ALAT, alanine aminotransferase; ASAT, aspartate aminotransferase; GGT, γ-glutamytransferase; PM2.5, atmospheric particulate matter with a diameter <2.5 μm

Of all risk variables, HbA_1c_ attained the highest HR (3.65 for 1 SD increase). Next, we ‘contextualised’ the individual HRs with respect to HbA_1c_ or estimated the equivalence of risk factors to HbA_1c_. The number of SDs of other continuous risk variables with respect to 1 SD increase of HbA_1c_ is presented in Fig. 3a, as well as the population mean and the value corresponding to the SD increase (Fig. 3b). The HRs adjusted for HbA_1c_ are depicted in Fig. 3c. Recall that a 1 SD increase in HbA_1c_ equates to 3.39 mmol/mol (0.31%) HbA_1c_. First, we observed that serum glucose is on par with HbA_1c_. Specifically, the HR for a 1 SD increase in HbA_1c_ is equivalent to an HR for a 1.08 SD (0.53 mmol/l; adjusted HR: 3.01) increase in glucose. Adiposity and HDL required at least a 1.5 SD change, a significant fraction of the population. For example, the HbA_1c_ equivalence for waist circumference was an increase of +1.66 SD (19.8 cm; adjusted HR: 1.60) and 1.67 SD for HDL-cholesterol (decrease of 1.67 SD; 0.67 mmol/l). Of note, other adiposity-related anthropometrics (i.e., body weight, WHR, BMI) needed a respective increase of 1.87, 1.88, and 2.03 SDs (27.6 kg, 0.15 units, 8.34 kg/m^2^) to be equivalent to a 1 SD change in HbA_1c_. Apart from uric acid (+1.95 SD; 0.14 mmol/l), all other replicated risk variables were required to increase by at least 3 SDs to be equivalent to the HR for a 1 SD change of HbA_1c_. For example, a 3.04 SD increase in leucocyte count has a HR equivalent to 1 SD increase in HbA_1c_. Only 418 individuals (0.43% of the Lifelines population) had a leucocyte count this high.

### Impact of impaired fasting glucose on the risk factors for developing type 2 diabetes

After we excluded individuals with IFG (*n* = 3510, 586 complete cases), all initially replicated risk factors remained nominally significant (ESM Fig. 4a). Sublevels for ECG (pathological) and smoking (ex-smoker) lost significance. HRs decreased for family history of diabetes (−14%) and HbA_1c_ (−15%) and increased for omeprazole and individual levels of three quality-of-life indicators (+11 to 22%). When correcting for IFG status, we found HRs to weaken for 22 risk variables (ESM Fig. 4b). HRs decreased by more than 10% for glycaemic traits, erythrocyte indicators, uric acid, adiposity-related variables, pathological ECG, family history of diabetes, eight medications and social functioning. HRs, *p* values and changes (%) in respect to the main analysis are described in ESM Table 3.

### Correlation patterns between risk factors

Correlation patterns between replicated variables are presented in ESM Fig. 5. We found correlations between variables to cluster for white blood cells, red blood cells, liver enzymes, adiposity-related anthropometrics, BP, dietary and smoking variables and quality of life (rho: >0.5). HDL-cholesterol showed weak to moderate inverse correlations with adiposity-related anthropometrics and triacylglycerols (rho: −0.27 to −0.45). All correlations remained stable across age groups and sexes. Sex-specific negative correlations were found for medications and differed between age groups. The number of effective variables decreased with at least one variable for the quality of life, anthropometric and the lifestyle group (ESM Table 4).

### Risk prediction and interchangeability of variables in clinical contexts

**Fig. 4:**
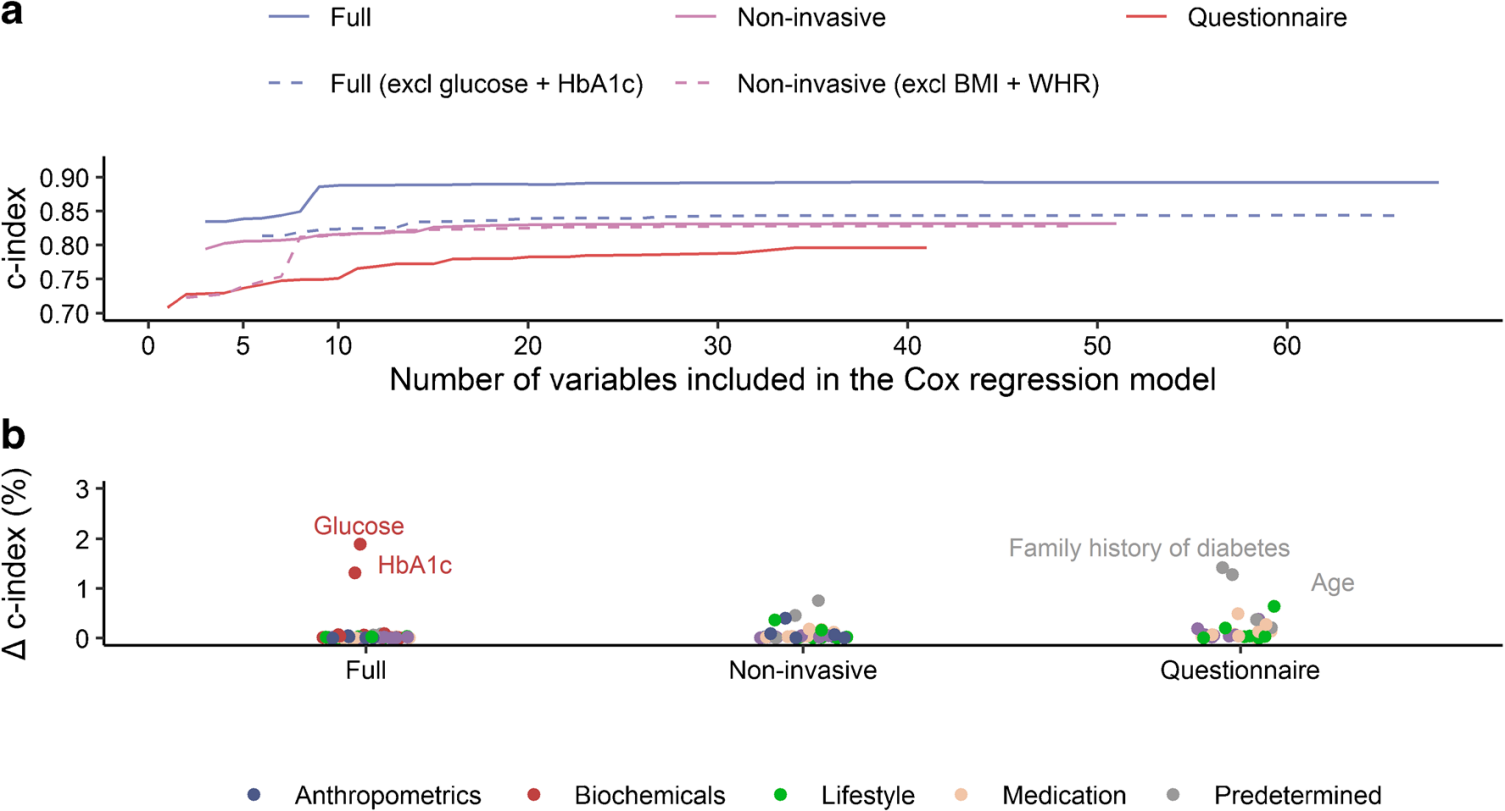
Applicability of risk variables for predicting type 2 diabetes. (**a**) The discrimination of prediction models for the development of type 2 diabetes containing an increasing number of risk variables. Models including non-invasive and invasive variables were satiated after four risk variables were included. Glucose and HbA_1c_ were solely responsible for the rise in discrimination between the full and non-invasive model, suggesting that other invasive variables do not contribute more to risk prediction than non-invasive variables do. Removing high scoring non-invasive measurements (i.e., BMI, WHR) appeared to lead to slightly larger models with similar discrimination, implying that these variables are more or less interchangeable. (**b**) Change in discrimination (c-index [%]) after the removal of one risk variable from the model containing all related variables including all variables, non-invasive variables and questionnaire variables. Differences of at least 1% were annotated

The number of times each variable was selected, the cumulative number of variables added to the model and the model’s corresponding c-index and HR are shown in Fig. 4a and reported in ESM Table 5. Impact is depicted in Fig. 4b and reported in ESM Table 6, and HR trajectories are shown in ESM Fig. 6.

When we included all replicated risk variables, HbA_1c_, HDL-cholesterol, and work-related activities were selected in all bootstrapped lasso regression models (c-index: 0.834). The next increase in c-index was observed after glucose was included (detected in 81% of the models, c-index: 0.886), after which the model satiated (c-index after all variables included: 0.892). The model’s c-index decreased when glucose (1.9%) or HbA_1c_ (1.3%) was removed. The inclusion of glucose decreased the HR of HDL-cholesterol (from 0.65 [0.60; 0.71] to 0.74 [0.68; 0.81]) and HbA_1c_ (from 3.44 [3.22; 3.68] to 2.05 [1.91; 2.20]). In contrast, the HR of male sex increased from 0.93 (0.80; 1.07) to 0.73 (0.63; 0.84).

To test the interchangeability of glucose and HbA_1c_, we excluded them as potential risk variables. This led the algorithm to select a more complex model with lower discrimination that included age, sex, BMI, HDL-cholesterol, triacylglycerols, the number of pack years and omeprazole, which scored at least 99% (c-index: 0.813 vs 0.886). The full model attained a c-index of 0.843 (vs 0.892) and was not impacted by individual variables (data not shown). We observed a similar increase in HR trajectory for sex (HR after inclusion of ten variables: from 0.97 [0.85; 1.11] to 0.67 [0.56; 0.79]).

Next, we excluded all invasive variables and ECG. We found the most robust scores (retained in 99% of the models) for age, BMI, WHR and omeprazole (c-index: 0.802). The full model attained a c-index of 0.831, and was borderline impacted by family history of diabetes (0.8%). The HR for age gradually became weaker over the inclusion of the first ten variables (from 2.09 [1.96; 2.22] to 1.70 [1.57; 1.83]), whereas HRs of other included variables remained stable over the inclusion process.

To investigate the interchangeability of the key variables BMI and WHR, we excluded these respective risk variables from the model. As a result, the algorithm selected more variables, including age, work-related activities, use of pantoprazole, omeprazole or simvastatin, heart rate, family history of diabetes and waist circumference, which resulted in a similar discrimination (c-index: 0.812) and remained stable as further variables were included (all variables: 0.828). The inclusion of waist circumference had an influence on the HR of age (2.03 [1.89; 2.19] to 1.84 [1.71; 1.99]), family history of diabetes (2.31 [2.04; 2.62] to 2.04 [1.80; 2.31]) and simvastatin, pantoprazole and omeprazole (range from 2.00–2.16 to 1.62–1.87).

When solely considering questionnaire-based variables (including age), then omeprazole, work-related activities and vigorous intensity activities were significant in 96% of bootstrapped models (c-index: 0.729). After adding sex, vitality, education, and pantoprazole (≥68% score), the c-index increased to 0.749. The full model reached a c-index of 0.796, which was impacted by age (1.3%) and family history of diabetes (1.4%). When more variables were added to the model, HRs declined for age (from 2.17 [2.07; 2.30] to 1.59 [1.46; 1.75]) and omeprazole (from 2.09 [1.76; 2.50] to 1.63 [1.35; 1.97]).

## Discussion

Here, we used a data-driven RV-WAS approach to systematically assess associations between 134 risk variables (to our knowledge the largest set to date) and the 5-year development of type 2 diabetes. We were able to identify, replicate and contextualise 63 risk variables robust to IFG. Next, we assessed their correlation and applicability in clinical risk prediction models using bootstrapped and cross-validated lasso-based linear regression models.

### Identification of risk variables for type 2 diabetes

Over the past decades, a plethora of different risk variables have been reported for the development of type 2 diabetes. By applying a RV-WAS approach to a population-based cohort, we screened potential risk variables in one cohort while accounting for multiple testing and subsequently replicated significant variables in a second, independent dataset. We identified a similar proportion of variables as a recent umbrella review of meta-analyses (47% vs 32%) [1], and the majority of identified risk variables have been described previously [1, 2, 4]. However, to the best of our knowledge, the prescription of proton-pump-inhibitors and quality of life have not been reported as risk variables for type 2 diabetes development before. Further, we found novel health-related quality-of-life variables that predict type 2 diabetes. These variables indicate the potential value of personal health perception on disease development.

### Risk variables put into context

Many identified risk variables showed relatively small HRs, such as inflammation and liver biomarkers. When we calculated how many SDs were needed to attain the same hazard as 1 SD increase in HbA_1c_ association, we found that 11 out of 23 biochemical variables were associated with a difference of at least 7 SDs, which is physiologically extreme, and adjusted HRs often attenuated to 1.00. When considering that 3 SDs were needed to increase HbA_1c_ concentrations from the population mean to the cut-off for diabetes, only glucose would be able to approximate a similar risk on its own. Therefore, although statistical significance may be important for aetiological investigation, these variables do not have clinically significant associations in risk. Interestingly, HRs for several lifestyle variables were larger compared with biochemical variables, suggesting that much debated food questionnaires are in fact on par with biochemical variables. In future studies, we will attempt to replicate the identified risk variables in an independent study population.

### Contextualisation of risk variables in clinical risk prediction models

The difference in discrimination between the full model and the non-invasive model were largely due to glycaemic variables. Some variables can now be seen in a new light, such as the difference between household-level and specific environmental factors. We found having a positive family history of diabetes to be uniquely represented in the non-invasive and questionnaire models. Family history contains information on both genetics and shared household environment or lifestyle [18]. In contrast, behaviours, such as smoking, in the presence of family history, may be indicative of individual and not shared exposure. In fact, smoking is represented in 70–85% of the clinical models as the risk variable ‘number of pack years’ and explains disease risk independent of its categorical counterpart.

Our data suggests that, prior to overt type 2 diabetes, many individuals were already being treated for complications of diabetes, such as CVDs. So far, medication has been sparsely used in risk prediction and limited to antihypertensive medication [4]. We identified the medications simvastatin, omeprazole and pantoprazole as robust risk variables. Interestingly, our method identified previously unrecognised questionnaire-based risk variables such as the health-related quality-of-life marker ‘physical functioning’. This variable was selected more often than established variables including indicators of energy intake, macronutrients and physical activity domains.

Further, we observed that the contribution of each variable in a model is co-dependent on the other variables in the model. For example, the HRs of HbA_1c_ and HDL-cholesterol decreased in the full model when glucose was added. A similar effect was seen when waist circumference was added to the non-invasive model without BMI and WHR. Comparing effect sizes and prediction across studies only makes sense if adjustment is made for the same or similar factors.

### Interchangeability of risk variables

Because only summarised data is available, it is impossible to assess correlations between variables in a meta-analysis. We uncovered an underlying tension between the (1) correlation pattern and (2) correlation size of risk variables. For example, (1) the observed correlations demonstrate a clustering pattern that can potentially be explained by their physiological origin; however, (2) the majority of correlations are modest to moderate (<0.5). Therefore, a priori selection of these variables in a risk model without extensive multivariate approaches makes it a challenge to discover their interchangeability and generalisability. This is exemplified in the clinical risk prediction models, which showed that most risk variables, albeit significant in the univariate analysis, did not contribute to risk prediction in addition to a few robust variables.

### Implementation of risk variables in future prediction models

In our data-driven assessment of 134 variables in three clinical models, we were not able to extensively outperform existing models [4]. In line with our findings, prediction models using a large number of omic measures, such as metabolites from a metabolomic assay, report many novel risk variables. However, when considering these variables in risk prediction next to established risk variables such as polygenic risk scores, BMI, glucose, smoking or physical activity, they add little incremental disease risk [19, 20, 21]. As evidenced by our data-driven approach, we believe that new discovery efforts should proceed with caution, as new models may show little incremental prediction benefit, albeit shining light on new aetiological paths.

In addition, the models reported here did not markedly improve after the inclusion of three of the most robust risk variables (HbA_1c_, HDL-cholesterol and work-related activities) and glucose. Externally validated prediction models for the development of type 2 diabetes include six to 13 risk variables [4]. These variables may contribute little or inconsistently to risk prediction and will need to be revisited to assess robust prediction across different cohorts. For example, omitting BMI and WHR from the non-invasive model led to a more complex model with similar discrimination. Using a systematic and data-driven approach can help to simplify and enhance generalisability of models for type 2 diabetes through transparent comparison of the interchangeability of potential variables.

### Limitations

Most Lifelines participants are white, so we were not able to reliably investigate associations with ethnicity. Moreover, this study is based on a Dutch population. The prevalence of diabetes in the Netherlands is similar to the average of other European countries (age-adjusted comparative prevalence: 5.4% vs 6.3%), yet lower than in the USA (10.8%) [22]. Also, some variables might be region specific. For example, it is common in the Netherlands to travel by bicycle (i.e., physical activity while commuting).

Further, we did not analyse some variables because of missingness. Although most of these variables were expensive and hard to obtain and therefore possibly not eligible for risk prediction in the first place, they may have been robust and unique risk variables.

### Conclusions

In conclusion, we demonstrated that a data-driven, RV-WAS method can be used to assess and contextualise a wide variety of potential risk variables for type 2 diabetes. Starting with 134 variables, we were able to identify 63 risk variables for the 5-year development of type 2 diabetes. However, we found that HRs for many replicated variables are negligible, leaving a small set of relevant variables. Moreover, only a small proportion of risk variables explain disease risk in a robust and unique fashion in prediction models for the development of type 2 diabetes. Adding variables to a satiated model can impact the HRs of those already included in the model. Therefore, association sizes of risk variables should only be compared across studies when models include the same or similar variables. We recommend a systematic approach in the assessment, contextualisation and clinical implementation of risk variables that are sensitive to the complex aetiology of the disease.

## Supplementary Information

ESM (PDF 4732 kb)

## Data Availability

Statistical code is available in the LIFEWAS package [17]. The manuscript is based on data from the Lifelines Cohort Study. Lifelines adheres to standards for data availability. The data catalogue of Lifelines is publicly accessible at www.lifelines.nl. All international researchers can obtain data at the Lifelines research office (research@lifelines.nl), for which a fee is required. The Lifelines system allows access for reproducibility of the study results.

## Abbreviations

FDR: False discovery rate
IFG: Impaired fasting glucose
RV-WAS: Risk variable-wide association study
UMCG: University Medical Center Groningen
