# Supplementary material for: Data-driven assessment, contextualisation and implementation of 134 variables in the risk for type 2 diabetes: an analysis of Lifelines, a prospective cohort study in the Netherlands
Source: Diabetologia. 2021 Mar 12;64(6):1268–78. doi: 10.1007/s00125-021-05419-1 (PMC8099846; doi:10.1007/s00125-021-05419-1)
Supplement: Supplementary file 1 — (PDF 4732 kb) [file 125_2021_5419_MOESM1_ESM.pdf]

Data-driven assessment and contextualization of 134 variables in their risk for type 2 diabetes: An analysis of Lifelines, a prospective cohort study in the Netherlands

T.P. van der Meer, B.H.R. Wolffenbuttel, C.J. Patel

ESM table 1. Characteristics of study population.

	Total population	Complete cases (n > 79,000)	Diabetics	
**General**				
N	96534	64392	1494	
Age	45.17 (12.59)	45.02 (12.14)	52.47 (11.67)	years
Sex = Male	39718 (41.1)	26727 (41.5)	763 (51.1)	
Diabetes status	1494 (1.5)	910 (1.4)	1494 (100.0)	
**Air pollution***				
NO2	21.36 (5.14)	21.35 (5.10)	21.35 (5.50)	units
PM10	24.10 (1.10)	24.09 (1.09)	24.08 (1.14)	units
PM2.5	15.62 (0.37)	15.62 (0.37)	15.63 (0.41)	units
**Alcohol, smoking****				
Alcohol (dietary)	6.75 (8.57)	6.81 (8.55)	7.24 (9.93)	g/day
Alcohol	12.50 (9.15)	12.44 (9.03)	13.41 (9.77)	n/month
Alcohol				days/month
0	18894 (20.1)	12546 (19.5)	360 (25.3)	
1	5857 (6.2)	3938 (6.1)	91 (6.4)	
2.5	12231 (13.0)	8311 (12.9)	170 (11.9)	
4.35	13981 (14.8)	9534 (14.8)	180 (12.6)	
10.9	22757 (24.2)	15872 (24.7)	267 (18.8)	
19.6	9964 (10.6)	6875 (10.7)	154 (10.8)	
28.2	10516 (11.2)	7294 (11.3)	202 (14.2)	
Alcohol				n/day
1	14374 (20.7)	9936 (20.7)	181 (19.1)	
2	29986 (43.1)	20923 (43.5)	372 (39.3)	
3	11550 (16.6)	8039 (16.7)	200 (21.1)	
4	6119 (8.8)	4193 (8.7)	86 (9.1)	
5	2658 (3.8)	1802 (3.7)	44 (4.7)	
6	2040 (2.9)	1354 (2.8)	30 (3.2)	
7	630 (0.9)	428 (0.9)	6 (0.6)	
8	953 (1.4)	611 (1.3)	9 (1.0)	
9	143 (0.2)	98 (0.2)	2 (0.2)	
10	589 (0.8)	402 (0.8)	9 (1.0)	
11	39 (0.1)	22 (0.0)	1 (0.1)	
12	449 (0.6)	291 (0.6)	6 (0.6)	
Number of pack years smoked	5.84 (9.44)	5.76 (9.30)	11.19 (14.03)	

Smoking				
Current smoker	17866 (18.8)	11103 (17.2)	327 (22.2)	
Ex smoker	32448 (34.2)	22027 (34.2)	646 (43.8)	
Never smoker	44601 (47.0)	31262 (48.5)	502 (34.0)	
**Anthropometrics**				
Body weight	79.28 (14.77)	79.35 (14.63)	90.51 (16.97)	kg
Length	174.85 (9.36)	175.04 (9.34)	174.39 (9.50)	cm
Body Mass Index	25.88 (4.10)	25.84 (4.06)	29.71 (4.83)	kg/m^2
Waist circumference	89.77 (11.93)	89.69 (11.77)	101.32 (12.43)	cm
Waist-to-hip ratio	0.90 (0.08)	0.90 (0.08)	0.96 (0.08)	units
Heartbeat	70.88 (10.74)	70.74 (10.66)	72.49 (11.56)	beats/min
Diastolic blood pressure	73.84 (9.26)	73.85 (9.21)	77.91 (10.06)	mmHg
Mean arterial pressure	93.22 (10.03)	93.17 (9.97)	98.99 (11.16)	mmHg
Systolic blood pressure	125.49 (15.13)	125.36 (15.01)	134.53 (16.42)	mmHg
Electrocardiogram				
borderline	4553 (4.7)	3029 (4.7)	108 (7.2)	
normal	90091 (93.5)	60284 (93.6)	1301 (87.2)	
pathologic	1759 (1.8)	1079 (1.7)	83 (5.6)	
**Biochemicals**				
ALAT	22.95 (15.42)	22.92 (14.53)	30.28 (23.42)	U/l
Albumin (serum)	45.06 (2.40)	45.15 (2.38)	44.64 (2.33)	g/l
Albumin (urine)	5.25 (31.60)	4.85 (22.80)	14.42 (150.38)	mg/l
Alkaline phosphatase	61.57 (17.49)	61.29 (17.38)	68.40 (19.05)	U/l
Anti-CCP	2.25 (14.11)	2.06 (11.95)	1.90 (6.53)	U/ml
Apolipoprotein A	1.62 (0.28)	1.62 (0.27)	1.50 (0.26)	g/l
Apolipoprotein B	0.92 (0.24)	0.92 (0.24)	1.08 (0.24)	g/l
ASAT	24.20 (9.73)	24.12 (8.78)	26.68 (14.26)	U/l
Basophilic granulocytes	0.03 (0.02)	0.03 (0.02)	0.03 (0.02)	$10^9/l$
Basophilic granulocytes (%)	0.54 (0.33)	0.54 (0.33)	0.52 (0.31)	%
Calcium (serum)	2.28 (0.08)	2.28 (0.08)	2.28 (0.08)	mmol/l
Cholesterol	5.10 (1.00)	5.09 (0.99)	5.28 (1.05)	mmol/l
C-reacive protein	2.47 (4.50)	2.35 (4.20)	4.38 (7.51)	mg/l
Creatinine (serum)	73.66 (13.00)	73.76 (12.86)	75.25 (14.73)	umol/l
Creatinine (urine)	8.25 (4.02)	8.25 (3.99)	8.71 (3.96)	mmol/l
CTD	0.31 (6.36)	0.32 (7.54)	0.17 (0.21)	units
Eosinophyl granulocytes	0.18 (0.13)	0.18 (0.13)	0.20 (0.14)	$10^9/l$
Eosinophyl granulocytes (%)	3.09 (1.96)	3.09 (1.97)	3.07 (1.89)	%
Erythrocytes	4.71 (0.40)	4.71 (0.40)	4.82 (0.39)	$10^{12}/l$
FT3	5.22 (0.82)	5.21 (0.82)	5.26 (1.12)	pmol/l
FT4	15.78 (2.29)	15.83 (2.25)	15.42 (2.31)	pmol/l
Gamma-GT	25.58 (22.36)	25.29 (22.24)	36.60 (25.14)	U/l
Glucose	4.92 (0.49)	4.91 (0.49)	5.78 (0.65)	mmol/l

HbA_{1c}	5.51 (0.31)	5.50 (0.31)	5.91 (0.34)	%
HbA_{1c}	36.7 (3.39)	36.6 (3.38)	41.10 (3.72)	mmol/mol
HDL-cholesterol	1.50 (0.40)	1.50 (0.39)	1.28 (0.36)	mmol/l
Hematocrit	0.42 (0.03)	0.42 (0.03)	0.43 (0.03)	%
Hemoglobin	8.76 (0.79)	8.77 (0.79)	8.98 (0.81)	mmol/l
LDL-cholesterol	3.25 (0.91)	3.25 (0.90)	3.43 (0.95)	mmol/l
Leukocytes	1.76 (0.26)	1.75 (0.25)	1.87 (0.28)	10^9/l
Lymfocytes	2.00 (0.58)	1.99 (0.57)	2.17 (0.68)	10^9/l
Lymfocytes (%)	34.16 (7.66)	34.21 (7.59)	33.13 (7.73)	%
Monocytes	0.48 (0.15)	0.48 (0.15)	0.53 (0.17)	10^9/l
Monocytes (%)	8.15 (1.96)	8.15 (1.95)	8.15 (1.96)	%
Neutrophilic granulocytes	3.26 (1.19)	3.25 (1.18)	3.73 (1.36)	10^9/l
Neutrophilic granulocytes (%)	54.05 (8.30)	54.00 (8.24)	55.13 (8.27)	%
Phosphate	0.91 (0.17)	0.91 (0.17)	0.89 (0.17)	mmol/l
Platelets	249.17 (55.85)	248.57 (55.23)	250.95 (57.46)	10^9/l
Potassium	3.87 (0.31)	3.86 (0.31)	3.90 (0.31)	mmol/l
Sodium	141.76 (1.84)	141.80 (1.84)	141.70 (1.88)	mmol/l
Triglycerides	1.16 (0.76)	1.14 (0.74)	1.76 (1.47)	mmol/l
TSH	2.59 (4.58)	2.58 (3.54)	2.68 (2.42)	mU/l
Ureum	5.18 (1.26)	5.16 (1.24)	5.43 (1.40)	mmol/l
Uric acid	0.29 (0.07)	0.29 (0.07)	0.34 (0.07)	
Family history (= Yes)				
History of father with diabetes	9585 (10.5)	6776 (10.5)	248 (17.9)	
History of mother with diabetes	11563 (12.7)	8124 (12.6)	341 (24.6)	
History of sibling with diabetes	4356 (4.8)	3032 (4.7)	191 (13.8)	
Family history of diabetes	21993 (24.1)	15501 (24.1)	618 (44.7)	
Family history of cardiovascular disease	12439 (43.9)	8925 (44.3)	169 (53.8)	
**Medication (ATC-code)**				
Acetylsalicylic acid (B01AC06)	1197 (1.5)	895 (1.4)	58 (4.9)	
Atorvastatin (C10AA01)	971 (1.2)	730 (1.1)	59 (5.0)	
Carbasalate calcium (B01AC08)	1345 (1.7)	1004 (1.6)	64 (5.4)	
Desloratadine (R06AX27)	1681 (2.1)	1406 (2.2)	17 (1.4)	
Diclofenac (M01AB05)	1691 (2.1)	1319 (2.0)	46 (3.9)	
Enalapril (C09AA02)	1107 (1.4)	885 (1.4)	62 (5.2)	
Fluticasone (R01AD08)	1394 (1.8)	1182 (1.8)	27 (2.3)	
Formoterol-Budesonide (R03AK07)	1666 (2.1)	1326 (2.1)	43 (3.6)	
Hydrochlorothiazide (C03AA03)	2530 (3.2)	1990 (3.1)	138 (11.6)	
Intrauterine device (G02BA03)	3087 (3.9)	2652 (4.1)	16 (1.3)	
Levocetirizine (R06AE09)	1268 (1.6)	1036 (1.6)	22 (1.9)	
Levothyroxine (H03AA01)	2921 (3.7)	2348 (3.6)	65 (5.5)	
Macrogol (A06AD65)	980 (1.2)	780 (1.2)	25 (2.1)	

Metoprolol (C07AB02)	2921 (3.7)	2315 (3.6)	151 (12.7)	
Mometasone (R01AD09)	957 (1.2)	793 (1.2)	19 (1.6)	
Omeprazole (A02BC01)	4969 (6.3)	3898 (6.1)	183 (15.4)	
Oral contraceptives (G03AA07)	5939 (7.5)	4939 (7.7)	38 (3.2)	
Pantoprazole (A02BC02)	1181 (1.5)	926 (1.4)	48 (4.0)	
Paroxetine (N06AB05)	1267 (1.6)	1056 (1.6)	24 (2.0)	
Salbutamol (R03AC02)	2590 (3.3)	2086 (3.2)	55 (4.6)	
Salmeterol-Fluticasone (R03AK06)	1390 (1.8)	1056 (1.6)	43 (3.6)	
Simvastatin (C10AA01)	2543 (3.2)	1972 (3.1)	127 (10.7)	
Sumatriptan (N02CC01)	1029 (1.3)	883 (1.4)	6 (0.5)	
**Noise exposure***				
Noise during day	55.60 (3.24)	55.62 (3.24)	55.51 (3.37)	dBA/h
Noise during evening	51.86 (3.24)	51.87 (3.24)	51.77 (3.37)	dBA/h
Noise during night	46.78 (3.24)	46.80 (3.24)	46.69 (3.37)	dBA/h
General noise	56.25 (3.24)	56.26 (3.24)	56.16 (3.37)	dBA/h
**Dietary nutrients****				
Energy	7771.24 (3605.81)	7811.99 (3513.97)	7255.06 (3792.13)	kJ
Energy	1857.37 (861.81)	1867.11 (839.86)	1734.00 (906.34)	kcal
Animal-based proteins	40.00 (17.81)	40.18 (17.34)	39.54 (19.20)	g/day
Plant-based proteins	28.29 (14.09)	28.54 (13.83)	26.03 (14.75)	g/day
Proteins	68.18 (29.18)	68.62 (28.42)	65.48 (31.30)	g/day
Monosacharide carbohydrates	89.78 (49.72)	89.71 (48.37)	82.02 (50.77)	g/day
Polysacharide carbohydrates	118.22 (59.22)	119.24 (58.07)	107.67 (60.75)	g/day
Carbohydrates	207.98 (100.76)	208.93 (98.32)	189.68 (102.84)	g/day
Fat	73.53 (38.13)	73.91 (37.17)	69.11 (40.52)	g/day
Coffee	28.86 (14.54)	29.00 (14.40)	30.64 (15.49)	n/month
**Physical activity****				
Household activities	1831.80 (1909.33)	1819.63 (1898.95)	1756.92 (1788.39)	units
Leisure activities	2517.98 (2453.82)	2525.14 (2433.84)	2750.73 (2648.99)	units
Work-related activities	3157.12 (3636.34)	3160.90 (3650.90)	3307.97 (4148.81)	units
Light intensity activity	3702.70 (2677.77)	3700.27 (2674.45)	3201.43 (2581.07)	units
Moderate intensity activity	2402.14 (3953.85)	2390.51 (3955.54)	3231.17 (4479.71)	units
Vigorous intensity activity	1748.72 (2037.47)	1764.89 (2038.36)	1644.03 (2141.35)	units
Watching television	144.10 (81.62)	143.68 (80.28)	180.03 (104.30)	min/day
**Health-related Quality of life**				
Bodily pain	84.84 (18.63)	85.52 (18.13)	80.59 (21.09)	units
Commuting	346.65 (682.85)	350.00 (687.17)	261.00 (598.36)	units
General health	72.69 (16.24)	73.38 (15.93)	67.32 (17.25)	units
Mental health	80.09 (13.36)	80.62 (12.92)	79.87 (14.53)	units
Physical functioning	91.11 (13.48)	91.51 (12.97)	84.21 (18.02)	units

Role emotional functioning				
0	4337 (4.6)	2572 (4.0)	88 (6.2)	
33.3	3351 (3.5)	2103 (3.3)	53 (3.7)	
50	18 (0.0)	12 (0.0)	52 (3.6)	
66.7	4660 (4.9)	3056 (4.7)	NA	
100	82258 (86.9)	56622 (88.0)	1209 (84.5)	
Role physical functioning				
0	6028 (6.4)	3745 (5.8)	141 (9.9)	
25	3264 (3.4)	2056 (3.2)	69 (4.8)	
33.3	36 (0.0)	20 (0.0)	1 (0.1)	
50	3987 (4.2)	2492 (3.9)	75 (5.2)	
66.7	31 (0.0)	19 (0.0)	80 (5.6)	
75	5897 (6.2)	3834 (6.0)	111 (7.8)	
100	75379 (79.7)	52226 (81.1)	1033 (72.2)	
Social functioning				
0	196 (0.2)	107 (0.2)	6 (0.4)	
12.5	323 (0.3)	189 (0.3)	4 (0.3)	
25	764 (0.8)	425 (0.7)	19 (1.3)	
37.5	1378 (1.5)	792 (1.2)	32 (2.2)	
50	2798 (3.0)	1686 (2.6)	NA	
62.5	8467 (8.9)	5431 (8.4)	153 (10.7)	
75	10383 (11.0)	6794 (10.6)	163 (11.4)	
87.5	16865 (17.8)	11374 (17.7)	271 (18.9)	
100	53505 (56.5)	37594 (58.4)	731 (51.1)	
Vitality	68.45 (16.73)	69.10 (16.43)	65.95 (18.02)	
**Sleep quality****				
Epworth Sleepiness Scale				
Higher Normal Daytime Sleepiness	22258 (30.6)	15275 (30.2)	337 (32.1)	
Lower Normal Daytime Sleepiness	43818 (60.3)	30918 (61.0)	597 (56.9)	
Mild Excessive Daytime Sleepiness	3456 (4.8)	2365 (4.7)	63 (6.0)	
Moderate Excessive Daytime Sleepiness	2247 (3.1)	1525 (3.0)	36 (3.4)	
Severe Excessive Daytime Sleepiness	891 (1.2)	564 (1.1)	16 (1.5)	
Pittsburgh Sleep Quality Index = Good	63163 (74.2)	45370 (75.3)	842 (71.9)	
Social Jetlag	1.09 (0.77)	1.10 (0.76)	0.94 (0.83)	units
**Socioeconomic factors***				
Education				
High	29264 (30.4)	20481 (31.8)	295 (19.9)	
Low	29506 (30.6)	18651 (29.0)	718 (48.4)	

Medium	37516 (39.0)	25260 (39.2)	470 (31.7)	
Income				
<750	3349 (4.1)	2054 (3.7)	21 (1.9)	euro/month
>3500	15841 (19.6)	11296 (20.4)	188 (16.8)	euro/month
1000-1500	7324 (9.0)	4689 (8.4)	128 (11.4)	euro/month
1500-2000	11803 (14.6)	7863 (14.2)	209 (18.6)	euro/month
2000-2500	13301 (16.4)	9120 (16.4)	218 (19.4)	euro/month
2500-3000	14696 (18.1)	10311 (18.6)	196 (17.5)	euro/month
3000-3500	12278 (15.2)	8691 (15.7)	118 (10.5)	euro/month
750-1000	2428 (3.0)	1478 (2.7)	44 (3.9)	euro/month
Race				
Arabic	254 (0.3)	176 (0.3)	4 (0.3)	
Asian	352 (0.4)	240 (0.4)	6 (0.5)	
Black	114 (0.1)	76 (0.1)	4 (0.3)	
European	83962 (98.3)	59476 (98.4)	1288 (97.5)	
Other	764 (0.9)	484 (0.8)	19 (1.4)	
Type of work				
Blue Semi	22748 (24.7)	14256 (23.1)	373 (29.3)	
Blue Skilled	8019 (8.7)	5171 (8.4)	160 (12.6)	
White Prof	26018 (28.2)	18183 (29.5)	313 (24.6)	
White Semi	35495 (38.5)	24124 (39.1)	425 (33.4)	
**Vitamins****				
Calcium (supplement)	8.36 (6.01)	8.53 (6.54)	8.10 (3.35)	n/month
Fish oil	7.99 (4.36)	7.95 (4.39)	8.09 (4.09)	n/month
Multivitamins (preparation)	27.36 (25.99)	26.96 (25.63)	32.62 (32.56)	n/month
Multivitamins (supplement)	8.40 (6.86)	8.11 (6.09)	6.65 (2.41)	n/month
Vitamin A/AD	8.97 (7.20)	9.02 (7.43)	8.04 (5.61)	n/month
Vitamin B	22.77 (19.54)	22.47 (18.49)	23.12 (12.86)	n/month
Vitamin C	8.39 (5.91)	8.42 (5.97)	8.84 (5.75)	n/month

Variables are expressed as mean (standard deviation) or number detected (%). Triglycerides was log-transformed in order to adjust for skewed distribution. *Grouped as pre-determined risk variables. **Grouped as lifestyle variables.

ESM table 2. Hazard ratios of replicated risk variables in the development of Type 2 Diabetes.

Risk variable	level	Hazard ratio (95% ci)	*p*-value	Complete cases (n)	Significant in n models
ALAT		1.1 (1.09; 1.12)	3.91E-28	42955	two
Albumin (serum)*		0.85 (0.77; 0.93)	3.35E-05	42954	one
Alkaline phosphatase		1.16 (1.13; 1.19)	4.65E-22	42955	two
Animal-based proteins (dietary)		1.28 (1.24; 1.33)	1.22E-24	94240	two
ASAT		1.06 (1.03; 1.08)	1.10E-05	42955	two
Atorvastatin	Yes	2.3 (2.03; 2.56)	1.02E-09	79274	two

Basophilic granulocytes (percentage)		0.87 (0.82; 0.93)	2.02E-06	94150	two
Bodily pain		0.81 (0.76; 0.86)	4.43E-19	94684	two
Body mass index		1.89 (1.86; 1.93)	1.48E-291	96511	two
C-reactive protein		1.1 (1.09; 1.12)	7.16E-32	42081	two
Creatinine (urine)		1.32 (1.26; 1.37)	1.34E-22	95751	two
Diastolic blood pressure		1.32 (1.27; 1.37)	4.60E-29	96490	two
Education	Low	1.81 (1.67; 1.95)	7.23E-17	96286	two
Education	Medium	1.34 (1.19; 1.48)	9.60E-05	96286	two
Electrocardiogram	borderline	1.51 (1.31; 1.71)	4.50E-05	96403	two
Electrocardiogram	pathologic	1.73 (1.5; 1.96)	2.47E-06	96403	two
Enalapril*	Yes	2.26 (2; 2.52)	8.46E-10	79274	one
Eosinophil granulocytes*		1.1 (1.06; 1.14)	2.47E-06	94151	one
Erythrocytes		1.38 (1.32; 1.43)	7.48E-26	95623	two
Family history of diabetes	Yes	2.39 (2.28; 2.49)	6.50E-58	91260	two
Fat (dietary)		1.25 (1.2; 1.3)	2.35E-19	94240	two
Gamma-GT		1.09 (1.08; 1.11)	7.50E-26	42954	two
General health		0.75 (0.7; 0.8)	1.08E-30	94661	two
Glucose		3.3 (3.26; 3.34)	0	95419	two
HbA1c		3.65 (3.59; 3.71)	0	95415	two
HDL-cholesterol		0.46 (0.4; 0.53)	1.50E-108	95823	two
Heartbeat		1.22 (1.17; 1.27)	1.85E-15	96476	two
Hematocrit		1.45 (1.39; 1.52)	4.01E-29	95624	two
Hemoglobin		1.41 (1.34; 1.48)	1.56E-22	95627	two
History of father with diabetes	Yes	2.05 (1.92; 2.19)	1.45E-24	91260	two
History of mother with diabetes	Yes	2.1 (1.98; 2.22)	1.76E-32	91260	two
History of sibling with diabetes	Yes	2.38 (2.23; 2.54)	6.38E-28	91260	two
Hydrochlorothiazide	Yes	2.54 (2.36; 2.73)	8.49E-23	79274	two
Leisure activities		0.86 (0.8; 0.92)	5.41E-07	87416	two
Leukocytes		1.53 (1.49; 1.57)	1.39E-85	95628	two
Lymphocytes		1.37 (1.32; 1.41)	1.43E-43	94150	two
Mean arterial pressure		1.37 (1.33; 1.42)	1.04E-43	96482	two
Metoprolol	Yes	2.38 (2.2; 2.56)	1.62E-21	79274	two
Monocytes		1.32 (1.28; 1.36)	8.87E-36	94150	two
Neutrophilic granulocytes		1.35 (1.31; 1.38)	4.30E-64	94150	two
Neutrophilic granulocytes (percentage)		1.14 (1.09; 1.2)	5.42E-07	94149	two
Omeprazole	Yes	2.17 (2.01; 2.33)	9.33E-21	79274	two
Packyears (smoking)		1.25 (1.22; 1.29)	6.35E-38	91976	two
Pantoprazole*	Yes	2.07 (1.77; 2.36)	1.03E-06	79274	one
Physical functioning		0.76 (0.72; 0.8)	6.26E-51	94666	two
Platelets*		1.11 (1.06; 1.15)	5.33E-05	95558	one
PM2.5*		1.15 (1.09; 1.21)	4.38E-06	57458	one

Proteins (dietary)		1.28 (1.23; 1.33)	4.72E-23	94240	two
Role emotional functioning	0	1.54 (1.32; 1.75)	0.000108456	94624	one
Role emotional functioning	33.33333333	1.27 (0.99; 1.54)	0.089893594	94624	one
Role emotional functioning	50	0 (-967.74; 967.74)	0.982401474	94624	one
Role emotional functioning	66.66666667	1.21 (0.99; 1.44)	0.0921074	94624	one
Role physical functioning	0	1.73 (1.55; 1.9)	1.39E-09	94622	two
Role physical functioning	25	1.57 (1.32; 1.81)	0.000298068	94622	two
Role physical functioning	33.33333333	2.24 (0.28; 4.2)	0.419831933	94622	two
Role physical functioning	50	1.33 (1.1; 1.57)	0.016491873	94622	two
Role physical functioning	66.66666667	0 (-1094.8; 1094.8)	0.983255818	94622	two
Role physical functioning	75	1.33 (1.13; 1.52)	0.004634383	94622	two
Salbutamol*	Yes	1.79 (1.52; 2.06)	2.50E-05	79274	one
Salmeterol-Fluticasone*	Yes	1.86 (1.55; 2.16)	6.88E-05	79274	one
Simvastatin	Yes	2.2 (2.01; 2.39)	1.13E-15	79274	two
Smoking	Current smoker	1.56 (1.42; 1.7)	4.33E-10	94915	two
Smoking	Ex smoker	1.25 (1.14; 1.37)	0.000190027	94915	two
Social functioning	0	3.41 (2.61; 4.21)	0.002790599	94679	two
Social functioning	12.5	1.12 (0.13; 2.1)	0.827075687	94679	two
Social functioning	25	2.18 (1.72; 2.63)	0.000833361	94679	two
Social functioning	37.5	1.96 (1.61; 2.31)	0.000199077	94679	two
Social functioning	50	1.54 (1.26; 1.82)	0.00268553	94679	two
Social functioning	62.5	1.48 (1.31; 1.66)	1.09E-05	94679	two
Social functioning	75	1.23 (1.06; 1.4)	0.018364736	94679	two
Social functioning	87.5	1.21 (1.07; 1.35)	0.008789649	94679	two
Systolic blood pressure		1.39 (1.35; 1.44)	1.46E-45	96490	two
Triglycerides		1.18 (1.17; 1.2)	4.16E-195	95823	two
Uric acid		1.94 (1.87; 2.01)	2.89E-73	42954	two
Vigorous intensity activity		0.84 (0.78; 0.9)	4.37E-08	87416	two
Vitality		0.79 (0.74; 0.84)	2.95E-20	94674	two
Waist-to-hip ratio		1.99 (1.93; 2.04)	2.09E-141	96508	two
Waist circumference		2.18 (2.14; 2.22)	1.56E-274	96511	two
Watching television		1.2 (1.17; 1.24)	1.18E-24	46743	two
Weight		2 (1.96; 2.05)	2.14E-232	96511	two
Work-related activities*		0.88 (0.82; 0.94)	2.84E-05	87416	one

ESM table 3a. Impact of excluding individuals with Impaired Fasting Glucose (IFG) on the replicated risk variables in the development of Type 2 Diabetes.

Variable	level	HR (95% ci)	*p*-value	Difference in HR (%)
ALAT		1.1 (1.08; 1.13)	2.70E-14	0
Albumin (serum)		0.83 (0.73; 0.93)	0.00021064	-2.4

Alkaline phosphatase		1.16 (1.12; 1.2)	1.77E-12	0
ASAT		1.06 (1.03; 1.09)	0.00083535	0
Atorvastatin	Yes	2.41 (2.02; 2.79)	6.10E-06	4.8
Body Mass Index		1.79 (1.74; 1.84)	5.46E-119	-5.3
C-reactive protein		1.09 (1.06; 1.12)	2.10E-10	-0.9
Diastolic blood pressure		1.21 (1.14; 1.28)	5.14E-08	-8.3
History of father with diabetes	Yes	1.83 (1.63; 2.02)	6.99E-10	-10.7
History of mother with diabetes	Yes	1.81 (1.64; 1.98)	1.68E-11	-13.8
History of sibling with diabetes	Yes	2.28 (2.06; 2.5)	1.85E-13	-4.2
Family history of diabetes	Yes	1.99 (1.84; 2.13)	1.78E-20	-16.7
Electrocardiogram	borderline	1.37 (1.08; 1.66)	0.031983339	-9.3
Education	Low	1.87 (1.69; 2.06)	4.62E-11	3.3
Education	Medium	1.27 (1.08; 1.47)	0.015521768	-5.2
Enalapril	Yes	2.04 (1.64; 2.43)	0.000384571	-9.7
Erythrocytes		1.31 (1.23; 1.39)	1.41E-10	-5.1
Fat (dietary)		1.29 (1.23; 1.35)	5.01E-15	3.2
Gamma-GT		1.09 (1.07; 1.11)	5.06E-13	0
Glucose		3.15 (3.06; 3.24)	2.47E-142	-4.5
Basophilic granulocytes (%)		0.9 (0.83; 0.98)	0.006942268	3.4
Eosinophil granulocytes		1.12 (1.07; 1.17)	4.13E-06	1.8
Neutrophilic granulocytes		1.33 (1.29; 1.38)	2.36E-32	-1.5
Neutrophilic granulocytes (%)		1.08 (1.01; 1.15)	0.038293892	-5.3
Heartbeat		1.14 (1.07; 1.2)	0.000190739	-6.6
HbA1c		3.11 (3.03; 3.19)	8.69E-181	-14.8
HDL-cholesterol		0.5 (0.41; 0.59)	1.11E-52	8.7
Hematocrit		1.4 (1.31; 1.48)	1.70E-13	-3.4
Hemoglobin		1.3 (1.2; 1.39)	5.29E-08	-7.8
Hydrochlorothiazide	Yes	2.46 (2.19; 2.73)	4.99E-11	-3.1
Vigorous intensity activity		0.83 (0.74; 0.91)	1.24E-05	-1.2
Creatinine (urine)		1.26 (1.19; 1.34)	1.35E-09	-4.5
Leisure activities		0.85 (0.77; 0.93)	0.000100296	-1.2
Leukocytes		1.56 (1.5; 1.61)	8.64E-54	2
Lymphocytes		1.42 (1.36; 1.48)	9.49E-33	3.6
Mean arterial pressure		1.27 (1.21; 1.34)	1.21E-13	-7.3
Metoprolol	Yes	2.44 (2.19; 2.69)	3.45E-12	2.5
Monocytes		1.37 (1.31; 1.42)	3.94E-26	3.8
Omeprazole	Yes	2.4 (2.18; 2.62)	8.71E-15	10.6
Packyears (smoking)		1.27 (1.22; 1.32)	4.65E-21	1.6
Pantoprazole	Yes	2.17 (1.76; 2.57)	0.000173057	4.8
PM2.5		1.14 (1.07; 1.22)	0.000715381	-0.9
Animal-based proteins (dietary)		1.33 (1.27; 1.39)	7.89E-19	3.9

Proteins (dietary)		1.34 (1.27; 1.4)	2.02E-18	4.7
Bodily pain		0.81 (0.74; 0.87)	1.74E-11	0
General health		0.73 (0.67; 0.8)	6.92E-21	-2.7
Physical functioning		0.76 (0.71; 0.8)	8.61E-30	0
Role emotional functioning	0	1.64 (1.35; 1.92)	0.000646839	6.5
Role emotional functioning	33	1.43 (1.08; 1.78)	0.045180658	12.6
Role emotional functioning	67	1.48 (1.2; 1.76)	0.005712807	22.3
Role physical functioning	0	1.88 (1.65; 2.11)	1.06E-07	8.7
Role physical functioning	25	1.9 (1.6; 2.2)	2.75E-05	21
Role physical functioning	75	1.43 (1.17; 1.69)	0.006981731	7.5
Social functioning	0	3.48 (2.34; 4.62)	0.031543164	2.1
Social functioning	25	2.37 (1.77; 2.97)	0.004836506	8.7
Social functioning	37.5	2.13 (1.67; 2.59)	0.001326575	8.7
Social functioning	50	1.66 (1.29; 2.03)	0.007590055	7.8
Social functioning	62.5	1.67 (1.44; 1.9)	1.03E-05	12.8
Social functioning	75	1.38 (1.15; 1.6)	0.004993924	12.2
Social functioning	87.5	1.29 (1.1; 1.48)	0.007887985	6.6
Vitality		0.78 (0.71; 0.84)	1.41E-13	-1.3
Salbutamol	Yes	1.77 (1.41; 2.13)	0.001945433	-1.1
Salmeterol-Fluticasone	Yes	1.73 (1.29; 2.16)	0.013935707	-7
Systolic blood pressure		1.3 (1.23; 1.36)	2.99E-15	-6.5
Simvastatin	Yes	2.19 (1.92; 2.47)	2.87E-08	-0.5
Smoking	Current smoker	1.62 (1.44; 1.81)	1.45E-07	3.8
Platelets		1.16 (1.1; 1.21)	3.46E-07	4.5
Triglycerides		1.18 (1.17; 1.2)	1.87E-106	0
Watching television		1.21 (1.16; 1.25)	8.29E-17	0.8
Uric acid		1.87 (1.78; 1.97)	4.26E-37	-3.6
Waist circumference		2.01 (1.95; 2.07)	1.40E-108	-7.8
Weight		1.85 (1.78; 1.91)	3.91E-85	-7.5
Waist-to-hip ratio		1.83 (1.75; 1.9)	8.38E-57	-8
Work-related activities		0.87 (0.79; 0.95)	0.000703778	-1.1

ESM table 3b. Impact of additionally adjusting for Impaired Fasting Glucose (IFG) on the replicated risk variables in the development of Type 2 Diabetes.

Variable	level	HR (95% ci)	*p*-value	Difference in HR (%)
ALAT		1.11 (1.09; 1.13)	2.46E-20	0.7
Albumin (serum)		0.84 (0.77; 0.92)	9.28E-06	-0.9

Alkaline phosphatase		1.14 (1.09; 1.18)	1.87E-09	-2.1
ASAT		1.06 (1.03; 1.09)	2.54E-05	0.3
Atorvastatin	Yes	1.88 (1.62; 2.15)	3.17E-06	-18.2
Body Mass Index		1.59 (1.56; 1.63)	2.56E-138	-15.7
C-reactive protein		1.09 (1.07; 1.12)	7.13E-15	-0.6
Diastolic blood pressure		1.23 (1.18; 1.28)	2.17E-16	-6.9
History of father with diabetes	Yes	1.79 (1.66; 1.93)	1.09E-16	-12.5
History of mother with diabetes	Yes	1.63 (1.5; 1.75)	1.28E-14	-22.5
History of sibling with diabetes	Yes	1.76 (1.6; 1.91)	2.05E-12	-26.2
Family history of diabetes	Yes	1.93 (1.82; 2.03)	4.98E-33	-19.4
Electrocardiogram	borderline	1.41 (1.21; 1.6)	0.000742815	-6.8
Electrocardiogram	pathologic	1.53 (1.3; 1.75)	0.000277417	-11.8
Education	Low	1.66 (1.52; 1.8)	1.45E-12	-8.5
Education	Medium	1.28 (1.14; 1.43)	0.000826196	-4.3
Enalapril	Yes	1.93 (1.67; 2.19)	6.19E-07	-14.4
Erythrocytes		1.24 (1.18; 1.3)	4.94E-12	-10.1
Fat (dietary)		1.26 (1.22; 1.31)	1.18E-21	1.2
Gamma-GT		1.08 (1.06; 1.1)	3.01E-10	-0.9
Glucose		2.92 (2.85; 2.99)	6.10E-184	-11.6
Basophilic granulocytes (%)		0.92 (0.86; 0.97)	0.002828119	5.5
Eosinophil granulocytes		1.13 (1.09; 1.17)	1.23E-08	2.7
Neutrophilic granulocytes		1.25 (1.21; 1.29)	2.09E-28	-7.5
Neutrophilic granulocytes (%)		1.06 (1.01; 1.11)	0.036056475	-7.3
Heartbeat		1.12 (1.07; 1.17)	8.02E-06	-8.4
HbA1c		2.6 (2.54; 2.66)	7.33E-220	-28.7
HDL-cholesterol		0.56 (0.49; 0.63)	1.46E-63	22.1
Hematocrit		1.3 (1.24; 1.36)	1.72E-15	-10.4
Hemoglobin		1.24 (1.17; 1.31)	4.09E-10	-12.1
Hydrochlorothiazide	Yes	1.96 (1.77; 2.14)	9.86E-13	-22.9
Vigorous intensity activity		0.88 (0.82; 0.94)	1.17E-05	4.3
Creatinine (urine)		1.24 (1.18; 1.29)	1.23E-13	-6.3
Leisure activities		0.91 (0.85; 0.96)	0.000650807	5.5
Leukocytes		1.41 (1.36; 1.45)	1.04E-45	-8
Lymphocytes		1.28 (1.24; 1.33)	5.55E-27	-6.2
Mean arterial pressure		1.25 (1.21; 1.3)	7.11E-22	-8.6
Metoprolol	Yes	1.86 (1.68; 2.03)	8.74E-12	-22
Monocytes		1.23 (1.19; 1.28)	1.98E-20	-6.7
Omeprazole	Yes	1.7 (1.54; 1.86)	1.82E-10	-21.6
Packyears (smoking)		1.16 (1.13; 1.19)	2.36E-18	-7.2
Pantoprazole	Yes	1.81 (1.52; 2.1)	6.70E-05	-12.7
PM2.5		1.12 (1.05; 1.18)	0.00040745	-3

Animal-based proteins (dietary)		1.28 (1.23; 1.32)	1.59E-24	-0.3
Proteins (dietary)		1.3 (1.25; 1.35)	3.70E-26	1.4
Bodily pain		0.84 (0.79; 0.88)	1.19E-14	3.2
General health		0.78 (0.73; 0.83)	4.56E-23	4.2
Physical functioning		0.8 (0.77; 0.84)	1.48E-33	5.6
Role emotional functioning	0	1.45 (1.23; 1.66)	0.000852687	-6
Role physical functioning	0	1.58 (1.4; 1.76)	3.76E-07	-8.6
Role physical functioning	25	1.65 (1.4; 1.89)	6.35E-05	4.8
Role physical functioning	75	1.31 (1.11; 1.51)	0.006908816	-1.5
Social functioning	25	1.81 (1.35; 2.26)	0.01095378	-17.1
Social functioning	37.5	2.04 (1.68; 2.39)	8.31E-05	4
Social functioning	50	1.41 (1.13; 1.7)	0.015844021	-8.1
Social functioning	62.5	1.43 (1.25; 1.6)	7.50E-05	-3.7
Social functioning	75	1.23 (1.06; 1.4)	0.018098714	-0.1
Vitality		0.82 (0.78; 0.87)	7.07E-15	4.4
Salbutamol	Yes	1.87 (1.6; 2.15)	5.74E-06	4.7
Salmeterol-Fluticasone	Yes	1.61 (1.31; 1.92)	0.00217335	-13.2
Systolic blood pressure		1.26 (1.21; 1.3)	4.19E-22	-9.5
Simvastatin	Yes	1.78 (1.59; 1.98)	4.20E-09	-18.9
Smoking	Current smoker	1.44 (1.3; 1.58)	2.73E-07	-7.4
Smoking	Ex smoker	1.17 (1.05; 1.29)	0.009882087	-6.4
Platelets		1.1 (1.05; 1.15)	0.000406288	-1.3
Triglycerides		1.17 (1.16; 1.19)	5.57E-87	-0.7
Watching television		1.2 (1.15; 1.24)	4.99E-18	-0.4
Uric acid		1.61 (1.54; 1.69)	6.39E-37	-16.9
Waist circumference		1.76 (1.72; 1.81)	1.41E-138	-19.2
Weight		1.63 (1.59; 1.67)	3.82E-111	-18.5
Waist-to-hip ratio		1.76 (1.7; 1.82)	6.13E-76	-11.6
Work-related activities		0.87 (0.81; 0.93)	4.35E-06	-0.8

ESM table 4. Number of effective variables.

Group	Number of effective variables	Total number of variables
Biochemical	22.12	23
Anthropometrics	7.8	9
Lifestyle	8.28	9
Medication	9	9
Quality of life	6.4	7
Predetermined	5.65	6

ESM table 5. Robustness of risk variables in clinical relevant prediction models.

Risk variable	level	Number of times selected	Cumulative number of variables	c-index	Hazard ratio (95% ci)
**Full model**					
HbA1c		100	3	0.834	3.74 (3.53; 3.96)
HDL-cholesterol		100	3	0.834	0.57 (0.53; 0.61)
Work-related activities*		100	3	0.834	0.8 (0.75; 0.84)
Sex	Male	98	4	0.835	1.13 (1; 1.28)
Triglycerides		95	5	0.839	1.15 (1.12; 1.18)
Proteins (dietary)		85	6	0.839	1.21 (1.15; 1.27)
Packyears (smoking)		84	7	0.843	1.17 (1.13; 1.21)
Pantoprazole*	Yes	82	8	0.849	1.61 (1.16; 2.23)
Glucose		81	9	0.886	2.52 (2.38; 2.66)
Body mass index		70	10	0.888	1.17 (1.11; 1.23)
Waist-to-hip ratio		69	11	0.889	1.2 (1.09; 1.31)
Family history of diabetes	Yes	65	12	0.888	1.58 (1.39; 1.8)
Omeprazole	Yes	64	13	0.889	1.33 (1.11; 1.59)
Erythrocytes		60	14	0.889	1.04 (0.96; 1.12)
Platelets*		53	15	0.889	0.93 (0.86; 0.99)
Age		52	16	0.889	1.21 (1.11; 1.32)
Electrocardiogram	borderline	45	17	0.890	1.29 (1; 1.65)
Simvastatin	Yes	45	17	0.890	1.27 (1.02; 1.57)
Systolic blood pressure		45	17	0.890	1.06 (1; 1.13)
Heartbeat		44	20	0.890	0.96 (0.9; 1.03)
Animal-based proteins (dietary)		42	21	0.890	0.88 (0.73; 1.06)
Hemoglobin		40	22	0.891	1.28 (1.14; 1.44)
Monocytes		33	23	0.891	1.1 (1.03; 1.17)
Hematocrit		30	24	0.891	1.17 (0.95; 1.44)
Diastolic blood pressure		29	25	0.891	1.03 (0.95; 1.13)
Mean arterial pressure		27	26	0.892	0.74 (0.56; 0.99)

Role emotional functioning	0	27	26	0.892	1.13 (0.84; 1.5)
Social functioning	0	25	28	0.892	0.58 (0.2; 1.65)
Education	Low	23	29	0.892	1.14 (0.95; 1.36)
Enalapril*	Yes	22	30	0.892	1.24 (0.92; 1.68)
Bodily pain		20	31	0.892	0.94 (0.88; 1.01)
Electrocardiogram	pathologic	20	31	0.892	1.06 (0.8; 1.42)
Atorvastatin	Yes	16	33	0.893	1.55 (1.13; 2.12)
Eosinophil granulocytes*		16	33	0.893	1.05 (0.98; 1.12)
Metoprolol	Yes	16	33	0.893	1.15 (0.93; 1.41)
Salbutamol*	Yes	16	33	0.893	1.22 (0.89; 1.68)
Role physical functioning	0	14	37	0.893	0.98 (0.72; 1.34)
Social functioning	50	14	37	0.893	0.93 (0.63; 1.37)
Neutrophilic granulocytes (percentage)		13	39	0.893	1.05 (0.98; 1.12)
Social functioning	25	13	39	0.893	1.47 (0.76; 2.84)
Waist circumference		13	39	0.893	0.87 (0.72; 1.05)
Fat (dietary)		12	42	0.893	0.93 (0.79; 1.1)
Vitality		12	42	0.893	0.97 (0.9; 1.05)
Basophilic granulocytes (percentage)		11	44	0.893	0.96 (0.89; 1.04)
Education	Medium	11	44	0.893	1.06 (0.88; 1.27)
Neutrophilic granulocytes		11	44	0.893	0.99 (0.88; 1.1)
Role emotional functioning	66.66666667	11	44	0.893	0.95 (0.7; 1.28)
Vigorous intensity activity		11	44	0.893	0.97 (0.9; 1.04)
Creatinine (urine)		10	49	0.892	1 (0.92; 1.09)
Smoking	Ex-smoker	10	49	0.892	0.93 (0.78; 1.11)
Social functioning	37.5	10	49	0.892	1.07 (0.63; 1.8)
Social functioning	75	10	49	0.892	1.02 (0.82; 1.28)
Social functioning	62.5	10	49	0.892	1.02 (0.8; 1.31)
Role physical functioning	25	9	54	0.892	1.02 (0.72; 1.44)
Smoking	Current smoker	9	54	0.892	0.93 (0.73; 1.18)
Salmeterol-Fluticasone*	Yes	8	56	0.892	1.16 (0.8; 1.68)
Social functioning	87.5	8	56	0.892	1.08 (0.9; 1.29)
Role physical functioning	50	7	58	0.892	1.08 (0.79; 1.48)
General health		6	59	0.892	0.97 (0.89; 1.05)
Role physical functioning	75	6	59	0.892	1.03 (0.79; 1.34)
Social functioning	12.5	6	59	0.892	0.43 (0.11; 1.75)
Weight		6	59	0.892	1 (0.84; 1.19)
Physical functioning		5	63	0.892	1 (0.93; 1.08)
Hydrochlorothiazide	Yes	4	64	0.892	1.02 (0.82; 1.28)
Role emotional functioning	33.33333333	3	65	0.892	0.96 (0.66; 1.42)
Leisure activities		2	66	0.892	0.99 (0.9; 1.09)

Lymphocytes		1	67	0.892	1.04 (0.9; 1.2)
Leukocytes		0	68	0.892	1.05 (0.69; 1.58)
Role emotional functioning	50	0	68	0.892	0 (0; Inf)
Role physical functioning	66.66666667	0	68	0.892	0 (0; Inf)
Role physical functioning	33.33333333	0	68	0.892	0 (0; Inf)
**Non-invasive model**					
Age		100	3	0.794	2.09 (1.96; 2.22)
Body mass index		100	3	0.794	1.82 (1.75; 1.89)
Omeprazole	Yes	100	3	0.794	1.63 (1.39; 1.92)
Waist-to-hip ratio		99	4	0.802	1.47 (1.39; 1.56)
Work-related activities*		97	5	0.805	0.87 (0.81; 0.93)
Pantoprazole*	Yes	80	6	0.806	1.89 (1.38; 2.61)
Proteins (dietary)		78	7	0.807	1.25 (1.18; 1.33)
Simvastatin	Yes	75	8	0.809	1.79 (1.46; 2.21)
Packyears (smoking)		69	9	0.814	1.18 (1.13; 1.23)
Metoprolol	Yes	64	10	0.816	1.59 (1.31; 1.94)
Atorvastatin	Yes	62	11	0.817	1.78 (1.32; 2.4)
Heartbeat		59	12	0.817	1.13 (1.07; 1.21)
Systolic blood pressure		58	13	0.819	1.17 (1.11; 1.25)
Enalapril*	Yes	51	14	0.819	1.42 (1.06; 1.9)
Family history of diabetes	Yes	50	15	0.826	2.02 (1.77; 2.29)
Salbutamol*	Yes	39	16	0.827	1.41 (1.05; 1.91)
Vitality		39	16	0.827	0.92 (0.87; 0.98)
Hydrochlorothiazide	Yes	36	18	0.829	1.54 (1.24; 1.9)
Vigorous intensity activity		33	19	0.829	0.95 (0.89; 1.01)
Education	Low	32	20	0.830	1.13 (0.95; 1.34)
Smoking	Current smoker	32	20	0.830	1.09 (0.87; 1.35)
Sex	Male	21	22	0.830	0.92 (0.77; 1.1)
Diastolic blood pressure		19	23	0.830	1.02 (0.94; 1.11)
Smoking	Ex-smoker	19	23	0.830	0.94 (0.79; 1.11)
General health		14	25	0.830	0.97 (0.9; 1.05)
Role physical functioning	0	14	25	0.830	0.92 (0.72; 1.18)
Animal-based proteins (dietary)		13	27	0.831	0.93 (0.77; 1.11)
Role emotional functioning	33.33333333	13	27	0.831	0.82 (0.56; 1.21)
Social functioning	12.5	13	27	0.831	0.33 (0.08; 1.38)
Fat (dietary)		12	30	0.831	0.97 (0.83; 1.14)
Role physical functioning	50	12	30	0.831	1.09 (0.82; 1.44)
Social functioning	75	12	30	0.831	0.94 (0.75; 1.18)
Social functioning	0	11	33	0.831	1.43 (0.51; 4.03)
Salmeterol-Fluticasone*	Yes	10	34	0.831	1.12 (0.78; 1.6)
Social functioning	50	9	35	0.831	0.84 (0.56; 1.25)

Bodily pain		8	36	0.831	0.99 (0.92; 1.07)
Education	Medium	8	36	0.831	1.04 (0.87; 1.25)
Mean arterial pressure		8	36	0.831	0.88 (0.65; 1.19)
Weight		7	39	0.831	1.02 (0.9; 1.17)
Role physical functioning	75	6	40	0.831	1.04 (0.81; 1.33)
Social functioning	87.5	6	40	0.831	1 (0.84; 1.2)
Social functioning	25	6	40	0.831	1.01 (0.53; 1.96)
Role emotional functioning	0	5	43	0.831	1.01 (0.73; 1.4)
Social functioning	37.5	5	43	0.831	0.96 (0.56; 1.64)
Waist circumference		5	43	0.831	1.02 (0.83; 1.24)
Role emotional functioning	66.66666667	4	46	0.831	0.93 (0.69; 1.27)
Physical functioning		3	47	0.831	0.97 (0.91; 1.05)
Role physical functioning	25	3	47	0.831	1 (0.72; 1.38)
Social functioning	62.5	3	47	0.831	1.01 (0.77; 1.32)
Leisure activities		1	50	0.831	0.95 (0.87; 1.05)
Role emotional functioning	50	0	51	0.831	0 (0; Inf)
Role physical functioning	66.66666667	0	51	0.831	0 (0; Inf)
Role physical functioning	33.33333333	0	51	0.831	0 (0; Inf)
**Questionnaire model**					
Age		100	1	0.708	2.18 (2.07; 2.3)
Omeprazole	Yes	98	2	0.727	2.09 (1.76; 2.5)
Vigorous intensity activity		98	2	0.727	0.86 (0.8; 0.92)
Work-related activities*		96	4	0.729	0.92 (0.87; 0.99)
Sex	Male	94	5	0.737	1.55 (1.37; 1.77)
Vitality		90	6	0.742	0.82 (0.78; 0.87)
Education	Low	88	7	0.747	1.68 (1.42; 1.98)
Pantoprazole*	Yes	69	8	0.749	2.01 (1.46; 2.77)
Education	Medium	59	9	0.749	1.3 (1.09; 1.54)
Bodily pain		43	10	0.751	0.9 (0.85; 0.96)
Family history of diabetes	Yes	37	11	0.765	2.32 (2.04; 2.63)
Simvastatin	Yes	35	12	0.769	1.96 (1.59; 2.43)
Enalapril*	Yes	33	13	0.772	1.88 (1.4; 2.51)
Proteins (dietary)		33	13	0.772	1.27 (1.19; 1.35)
Salbutamol*	Yes	31	15	0.772	1.66 (1.23; 2.23)
Packyears (smoking)		30	16	0.779	1.24 (1.19; 1.3)
Animal-based proteins (dietary)		25	17	0.780	1.12 (0.93; 1.34)
Smoking	Current smoker	21	18	0.780	0.92 (0.74; 1.14)
Smoking	Ex-smoker	17	19	0.780	0.88 (0.75; 1.04)
Atorvastatin	Yes	10	20	0.782	2.18 (1.61; 2.95)
General health		10	20	0.782	0.88 (0.82; 0.95)
Fat (dietary)		8	22	0.782	0.95 (0.8; 1.11)

Role emotional functioning	66.66666667	5	23	0.784	0.92 (0.68; 1.24)
Salmeterol-Fluticasone*	Yes	5	23	0.784	1.22 (0.85; 1.74)
Social functioning	0	5	23	0.784	1.05 (0.37; 2.99)
Role physical functioning	50	4	26	0.785	1.19 (0.88; 1.59)
Role physical functioning	0	4	26	0.785	0.88 (0.65; 1.18)
Social functioning	87.5	4	26	0.785	0.98 (0.82; 1.18)
Social functioning	37.5	4	26	0.785	0.84 (0.5; 1.42)
Social functioning	62.5	4	26	0.785	0.93 (0.71; 1.21)
Metoprolol	Yes	3	31	0.788	1.81 (1.48; 2.22)
Role emotional functioning	33.33333333	3	31	0.788	0.84 (0.57; 1.23)
Social functioning	75	3	31	0.788	0.89 (0.71; 1.12)
Hydrochlorothiazide	Yes	2	34	0.796	1.92 (1.56; 2.38)
Physical functioning		2	34	0.796	0.85 (0.79; 0.91)
Role physical functioning	75	2	34	0.796	1.04 (0.8; 1.34)
Social functioning	50	2	34	0.796	0.85 (0.57; 1.27)
Social functioning	25	2	34	0.796	0.95 (0.5; 1.84)
Role emotional functioning	0	1	39	0.796	1 (0.72; 1.39)
Role physical functioning	25	1	39	0.796	0.99 (0.7; 1.38)
Leisure activities		0	41	0.796	0.93 (0.84; 1.02)
Role emotional functioning	50	0	41	0.796	0 (0; Inf)
Role physical functioning	66.66666667	0	41	0.796	0 (0; Inf)
Role physical functioning	33.33333333	0	41	0.796	0 (0; Inf)
Social functioning	12.5	0	41	0.796	0.32 (0.08; 1.35)
**Full model (excluding glycaemic traits)**					
Age		100	6	0.813	2.1 (1.97; 2.25)
Body mass index		100	6	0.813	1.67 (1.6; 1.74)
HDL-cholesterol		100	6	0.813	0.67 (0.62; 0.72)
Omeprazole	Yes	100	6	0.813	1.52 (1.28; 1.8)
Packyears (smoking)		100	6	0.813	1.18 (1.13; 1.23)
Triglycerides		100	6	0.813	1.14 (1.11; 1.17)
Sex	Male	99	7	0.813	0.97 (0.85; 1.11)
Work-related activities*		96	8	0.819	0.87 (0.81; 0.93)
Waist-to-hip ratio		92	9	0.822	1.34 (1.23; 1.45)
Proteins (dietary)		86	10	0.823	1.28 (1.21; 1.36)
Pantoprazole*	Yes	85	11	0.824	1.79 (1.29; 2.49)
Simvastatin	Yes	63	12	0.825	1.74 (1.41; 2.14)
Neutrophilic granulocytes		62	13	0.826	1.16 (1.1; 1.22)
Family history of diabetes	Yes	60	14	0.834	2 (1.76; 2.28)
Vitality		45	15	0.834	0.94 (0.88; 1)
Electrocardiogram	borderline	42	16	0.835	1.28 (1; 1.65)
Enalapril*	Yes	42	16	0.835	1.44 (1.06; 1.94)

Atorvastatin	Yes	40	18	0.836	1.76 (1.29; 2.4)
Systolic blood pressure		36	19	0.838	1.17 (1.1; 1.24)
Electrocardiogram	pathologic	33	20	0.839	1.25 (0.93; 1.69)
Hydrochlorothiazide	Yes	33	20	0.839	1.53 (1.24; 1.89)
Basophilic granulocytes (percentage)		29	22	0.839	0.94 (0.87; 1.01)
Salbutamol*	Yes	29	22	0.839	1.44 (1.05; 1.96)
Monocytes		28	24	0.840	1.06 (0.99; 1.14)
Creatinine (urine)		26	25	0.839	1.05 (0.97; 1.14)
Metoprolol	Yes	25	26	0.839	1.15 (0.93; 1.42)
Education	Low	24	27	0.842	1.1 (0.92; 1.31)
Hematocrit		24	27	0.842	1.16 (1.07; 1.26)
Role emotional functioning	33.33333333	22	29	0.842	0.75 (0.51; 1.12)
Neutrophilic granulocytes (percentage)		21	30	0.843	0.93 (0.84; 1.03)
Social functioning	75	19	31	0.843	0.91 (0.72; 1.15)
Platelets*		18	32	0.843	0.98 (0.91; 1.05)
Vigorous intensity activity		18	32	0.843	0.97 (0.9; 1.04)
Role emotional functioning	66.66666667	14	34	0.843	0.91 (0.67; 1.23)
Salmeterol-Fluticasone*	Yes	14	34	0.843	1.2 (0.83; 1.75)
Animal-based proteins (dietary)		13	36	0.843	0.93 (0.77; 1.12)
Erythrocytes		12	37	0.843	1.05 (0.93; 1.19)
General health		12	37	0.843	0.98 (0.91; 1.06)
Heartbeat		12	37	0.843	1.03 (0.97; 1.1)
Role physical functioning	0	12	37	0.843	0.91 (0.68; 1.2)
Social functioning	50	12	37	0.843	0.82 (0.55; 1.22)
Social functioning	87.5	10	42	0.843	0.97 (0.81; 1.16)
Diastolic blood pressure		9	43	0.843	1 (0.92; 1.09)
Leisure activities		9	43	0.843	0.96 (0.87; 1.06)
Role physical functioning	50	9	43	0.843	1.12 (0.83; 1.51)
Social functioning	0	9	43	0.843	1.41 (0.5; 3.94)
Bodily pain		8	47	0.843	1 (0.92; 1.08)
Role emotional functioning	0	8	47	0.843	0.99 (0.73; 1.34)
Social functioning	37.5	8	47	0.843	0.92 (0.55; 1.56)
Waist circumference		8	47	0.843	0.97 (0.82; 1.16)
Education	Medium	7	51	0.843	1.04 (0.86; 1.24)
Physical functioning		7	51	0.843	1.01 (0.94; 1.08)
Role physical functioning	75	7	51	0.843	1.07 (0.82; 1.39)
Smoking	Current smoker	7	51	0.843	0.88 (0.69; 1.11)
Hemoglobin		6	55	0.843	0.97 (0.8; 1.18)
Lymphocytes		6	55	0.843	1.03 (0.9; 1.18)

Role physical functioning	25	6	55	0.843	1.04 (0.74; 1.45)
Social functioning	25	6	55	0.843	0.94 (0.48; 1.84)
Social functioning	62.5	6	55	0.843	0.98 (0.75; 1.27)
Weight		6	55	0.843	1 (0.85; 1.18)
Mean arterial pressure		5	61	0.843	0.9 (0.62; 1.29)
Smoking	Ex-smoker	5	61	0.843	0.96 (0.81; 1.14)
Social functioning	12.5	5	61	0.843	0.41 (0.1; 1.7)
Eosinophil granulocytes*		3	64	0.843	1.01 (0.94; 1.09)
Fat (dietary)		1	65	0.843	0.97 (0.82; 1.14)
Leukocytes		0	66	0.843	1.23 (0.82; 1.84)
Role emotional functioning	50	0	66	0.843	0 (0; Inf)
Role physical functioning	66.66666667	0	66	0.843	0 (0; Inf)
Role physical functioning	33.33333333	0	66	0.843	0 (0; Inf)
**Non-invasive model (excluding BMI and WHR)**					
Age		100	2	0.722804033	2.13 (2.01; 2.26)
Omeprazole	Yes	100	2	0.722804033	2.13 (1.81; 2.5)
Work-related activities*		96	3	0.725284563	0.93 (0.87; 0.99)
Pantoprazole*	Yes	85	4	0.727786637	2.31 (1.68; 3.18)
Heartbeat		73	5	0.739269036	1.19 (1.12; 1.26)
Simvastatin	Yes	73	5	0.739269036	2.2 (1.78; 2.71)
Family history of diabetes	Yes	64	7	0.753421259	2.31 (2.04; 2.62)
Waist circumference		63	8	0.811870119	2.04 (1.94; 2.15)
Proteins (dietary)		62	9	0.812603559	1.26 (1.19; 1.34)
Systolic blood pressure		57	10	0.814730658	1.18 (1.11; 1.25)
Education	Low	55	11	0.816460106	1.25 (1.06; 1.48)
Vitality		54	12	0.817710011	0.9 (0.85; 0.96)
Enalapril*	Yes	53	13	0.820972819	1.47 (1.09; 1.97)
Packyears (smoking)		53	13	0.820972819	1.17 (1.12; 1.22)
Metoprolol	Yes	51	15	0.822422132	1.65 (1.35; 2.02)
Sex	Male	50	16	0.822832668	0.9 (0.77; 1.04)
Vigorous intensity activity		47	17	0.823093839	0.96 (0.89; 1.02)
Atorvastatin	Yes	43	18	0.824244597	1.91 (1.41; 2.59)
Salbutamol*	Yes	43	18	0.824244597	1.43 (1.06; 1.93)
Smoking	Current smoker	30	20	0.824433072	1.06 (0.85; 1.32)
Hydrochlorothiazide	Yes	27	21	0.826200309	1.59 (1.29; 1.96)
Diastolic blood pressure		25	22	0.82627271	1.02 (0.94; 1.11)
Weight		24	23	0.826279973	1.11 (0.98; 1.26)
Smoking	Ex-smoker	23	24	0.826279973	0.92 (0.78; 1.09)
General health		20	25	0.826256864	0.97 (0.9; 1.04)
Bodily pain		15	26	0.826259976	1 (0.93; 1.07)
Education	Medium	13	27	0.827229946	1.08 (0.91; 1.29)

Role emotional functioning	33.33333333	13	27	0.827229946	0.81 (0.55; 1.2)
Role physical functioning	0	13	27	0.827229946	0.92 (0.69; 1.24)
Social functioning	12.5	13	27	0.827229946	0.32 (0.08; 1.35)
Physical functioning		12	31	0.827470983	0.96 (0.9; 1.03)
Role physical functioning	50	12	31	0.827470983	1.09 (0.81; 1.46)
Social functioning	75	12	31	0.827470983	0.93 (0.74; 1.17)
Mean arterial pressure		11	34	0.827826921	0.89 (0.64; 1.23)
Salmeterol-Fluticasone*	Yes	11	34	0.827826921	1.12 (0.78; 1.62)
Fat (dietary)		10	36	0.827830832	0.99 (0.85; 1.15)
Animal-based proteins (dietary)		9	37	0.827727001	0.91 (0.75; 1.11)
Social functioning	50	8	38	0.827727001	0.86 (0.58; 1.3)
Social functioning	0	8	38	0.827727001	1.36 (0.48; 3.86)
Role emotional functioning	0	6	40	0.827727001	1.01 (0.73; 1.4)
Role physical functioning	75	6	40	0.827727001	1.04 (0.81; 1.35)
Role physical functioning	25	6	40	0.827727001	1 (0.71; 1.41)
Social functioning	62.5	5	43	0.827727001	0.99 (0.76; 1.3)
Social functioning	25	5	43	0.827727001	0.95 (0.49; 1.84)
Social functioning	87.5	5	43	0.827727001	1.01 (0.84; 1.2)
Role emotional functioning	66.66666667	4	46	0.827727001	0.95 (0.7; 1.28)
Social functioning	37.5	4	46	0.827727001	1.01 (0.59; 1.73)
Leisure activities		2	48	0.82777451	0.96 (0.87; 1.06)
Role emotional functioning	50	0	49	0.82777451	0 (0; Inf)
Role physical functioning	66.66666667	0	49	0.82777451	0 (0; Inf)
Role physical functioning	33.33333333	0	49	0.82777451	0 (0; Inf)

Robustness of variables was assessed by 100x bootstrapped and cross-validated Lasso-regression models. The column “Number of times selected” corresponds to the cumulative number of times a variable was selected by the lasso-regression and found to be significant ($p<0.05$). Next, we built a robust prediction model by stepwise including the most robust risk variables in the model using cox-regression. The model discrimination (c-index) and risk variable coefficient at the moment of inclusion was reported at each inclusion step.

Supplementary table 6. Impact of individual risk variables on clinically relevant prediction models**.**

Excluded variable	New C-index	Absolute difference (%)
**Full**		
Glucose	0.875	1.89
HbA1c	0.881	1.31
HDL-cholesterol	0.891	0.09

Family history of diabetes	0.893	0.08
Triglycerides	0.892	0.07
Sex	0.892	0.06
Creatinine (urine)	0.893	0.06
Body mass index	0.892	0.05
Platelets*	0.892	0.04
Erythrocytes	0.892	0.03
Proteins (dietary)	0.893	0.03
Simvastatin	0.892	0.03
Work-related activites*	0.892	0.03
Animal-based proteins (dietary)	0.892	0.03
Atorvastatin	0.892	0.03
Omeprazole	0.892	0.02
Systolic blood pressure	0.892	0.02
Smoking	0.892	0.02
Bodily pain	0.892	0.02
General health	0.892	0.02
Role emotional functioning	0.892	0.02
Neutrophilic granulocytes (percentage)	0.892	0.02
Diastolic blood pressure	0.892	0.01
Electrocardiogram	0.892	0.01
Mean arterial pressure	0.892	0.01
Packyears (smoking)	0.892	0.01
Neutrophilic granulocytes	0.892	0.01
Enalapril*	0.892	0.01
Education	0.892	0.01
Hemoglobin	0.892	0.01
Hematocrit	0.892	0.01
Social functioning	0.892	0.01
Basophilic granulocytes (percentage)	0.892	0.01
Waist-to-hip ratio	0.892	0.01
Vigorous intensity activity	0.892	0.01
Role physical functioning	0.892	0.01
Eosinophyl granulocytes*	0.892	0.01
Monocytes	0.892	0.00
Pantoprazole*	0.892	0.00
Hydrochlorothiazide	0.892	0.00
Salmeterol-Fluticasone*	0.892	0.00
Waist circumference	0.892	0.00
Salbumatol*	0.892	0.00
Heartbeat	0.892	0.00

Fat (dietary)	0.892	0.00
Lymfocytes	0.892	0.00
Metoprolol	0.892	0.00
Leukocytes	0.892	0.00
Age	0.892	0.00
Leisure activities	0.892	0.00
Physical functioning	0.892	0.00
Vitality	0.892	0.00
Weight	0.892	0.00
**Non-invasive**		
Family history of diabetes	0.821	0.80
Waist circumference	0.823	0.61
Age	0.824	0.52
Packyears (smoking)	0.825	0.36
Simvastatin	0.826	0.20
Hydrochlorothiazide	0.826	0.20
Work-related activites*	0.826	0.16
Atorvastatin	0.827	0.13
Education	0.827	0.10
Systolic blood pressure	0.827	0.07
Metoprolol	0.827	0.06
Omeprazole	0.827	0.05
Vitality	0.827	0.05
Role physical functioning	0.827	0.05
Role emotional functioning	0.827	0.05
Sex	0.828	0.03
Diastolic blood pressure	0.828	0.03
Pantoprazole*	0.828	0.03
Salmeterol-Fluticasone*	0.828	0.03
Smoking	0.828	0.03
Physical functioning	0.828	0.02
Salbumatol*	0.828	0.02
Social functioning	0.828	0.02
Enalapril*	0.828	0.02
Mean arterial pressure	0.828	0.02
Animal-based proteins (dietary)	0.828	0.01
General health	0.828	0.01
Vigorous intensity activity	0.828	0.01
Bodily pain	0.828	0.01
Proteins (dietary)	0.828	0.01
Leisure activities	0.828	0.01

Weight	0.828	0.01
Fat (dietary)	0.828	0.00
Heartbeat	0.828	0.00
**Questionnaire**		
Family history of diabetes	0.784	1.42
Age	0.785	1.28
Packyears (smoking)	0.791	0.64
Hydrochlorothiazide	0.792	0.49
Physical functioning	0.793	0.37
Sex	0.793	0.37
Simvastatin	0.793	0.27
Education	0.794	0.21
Work-related activites*	0.794	0.20
Role physical functioning	0.794	0.19
Atorvastatin	0.795	0.13
Metoprolol	0.795	0.13
Omeprazole	0.795	0.07
Social functioning	0.795	0.07
Salmeterol-Fluticasone*	0.795	0.06
Role emotional functioning	0.795	0.06
Vitality	0.795	0.05
General health	0.795	0.04
Salbumatol*	0.796	0.04
Vigorous intensity activity	0.795	0.04
Animal-based proteins (dietary)	0.795	0.03
Pantoprazole*	0.795	0.03
Proteins (dietary)	0.796	0.03
Bodily pain	0.795	0.02
Enalapril*	0.796	0.01
Leisure activities	0.796	0.00
Smoking	0.796	0.00
Fat (dietary)	0.796	0.00

ESM figure 1. Study population selection.

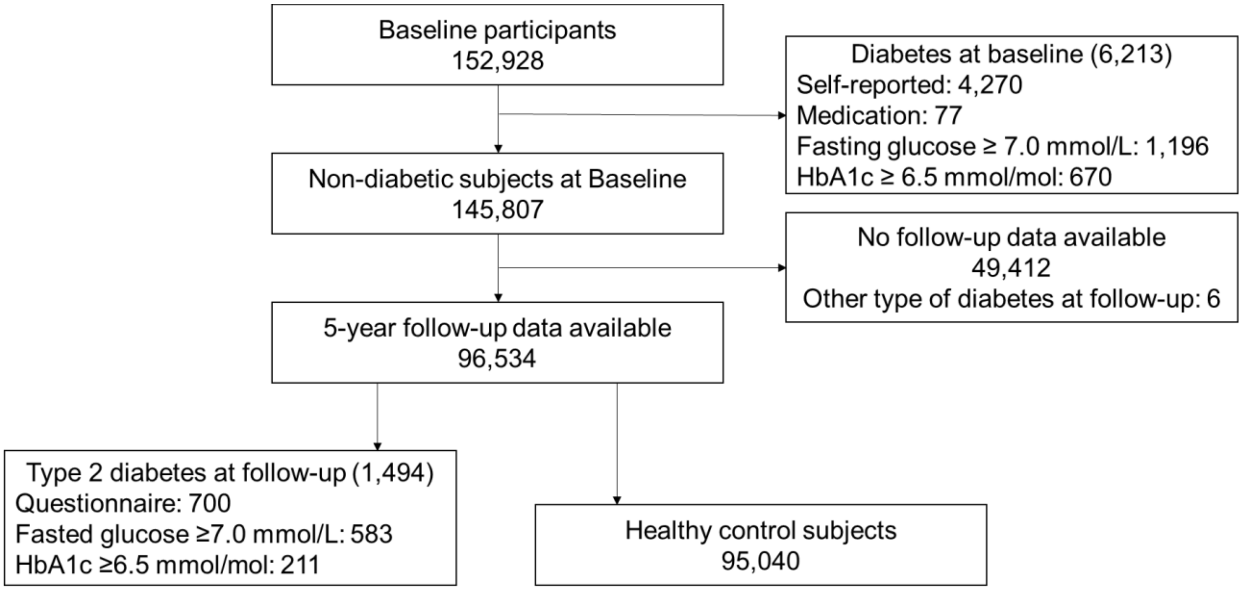

The Lifelines cohort study consists of 152,928 participants from the three most northern provinces of the Netherlands. First, all individuals with diabetes at baseline, individuals with no available follow-up data, or individuals which were diagnosed at follow-up with a type of diabetes other than Type 2 were excluded. Next, T2D cases were identified through questionnaire, fasting glucose levels or HbA1c levels, resulting in 1.494 cases (1.5%) and 95.040 controls.

ESM figure 2. Hazard ratios of replicated variables after excluding pre-diabetic indivuals using the ADA and WHO criteria.

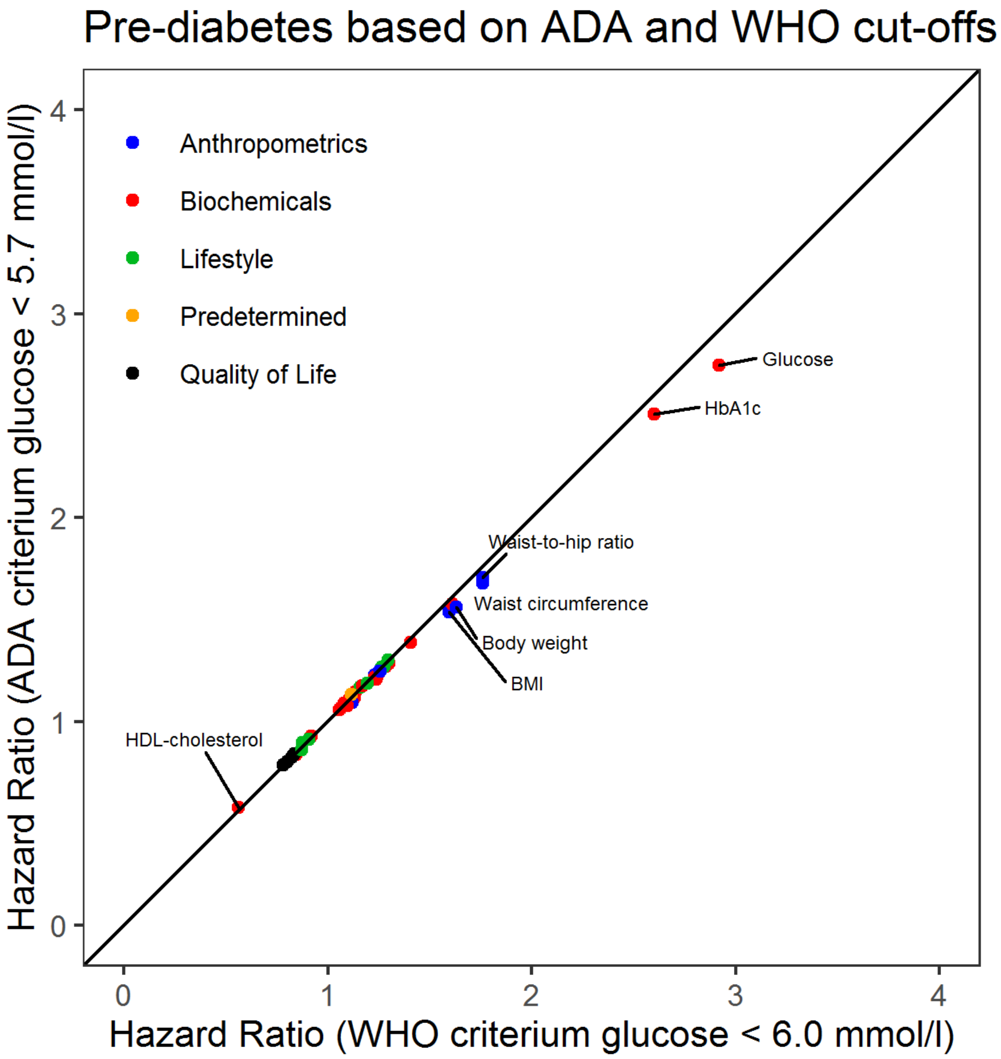

Every dot represents one replicated risk variable for the development of type 2 diabetes. The x-axis represents the hazard ratio a variable attained after the exclusion of pre-diabetic individuals using the WHO criterium of HbA_{1c} < 6.0 mmol/l, whereas the hazard ratios depicted on the y-axis were attained using the ADA criterium of HbA_{1c} < 5.7 mmol/l. All variables for which the hazard ratio differed for more than 2.5% between the criteria are annotated. The largest difference was for glucose (5.8% higher hazard ratio when using the WHO criterium).

ESM Figure 3a. Volcano plots of replicated risk variables.

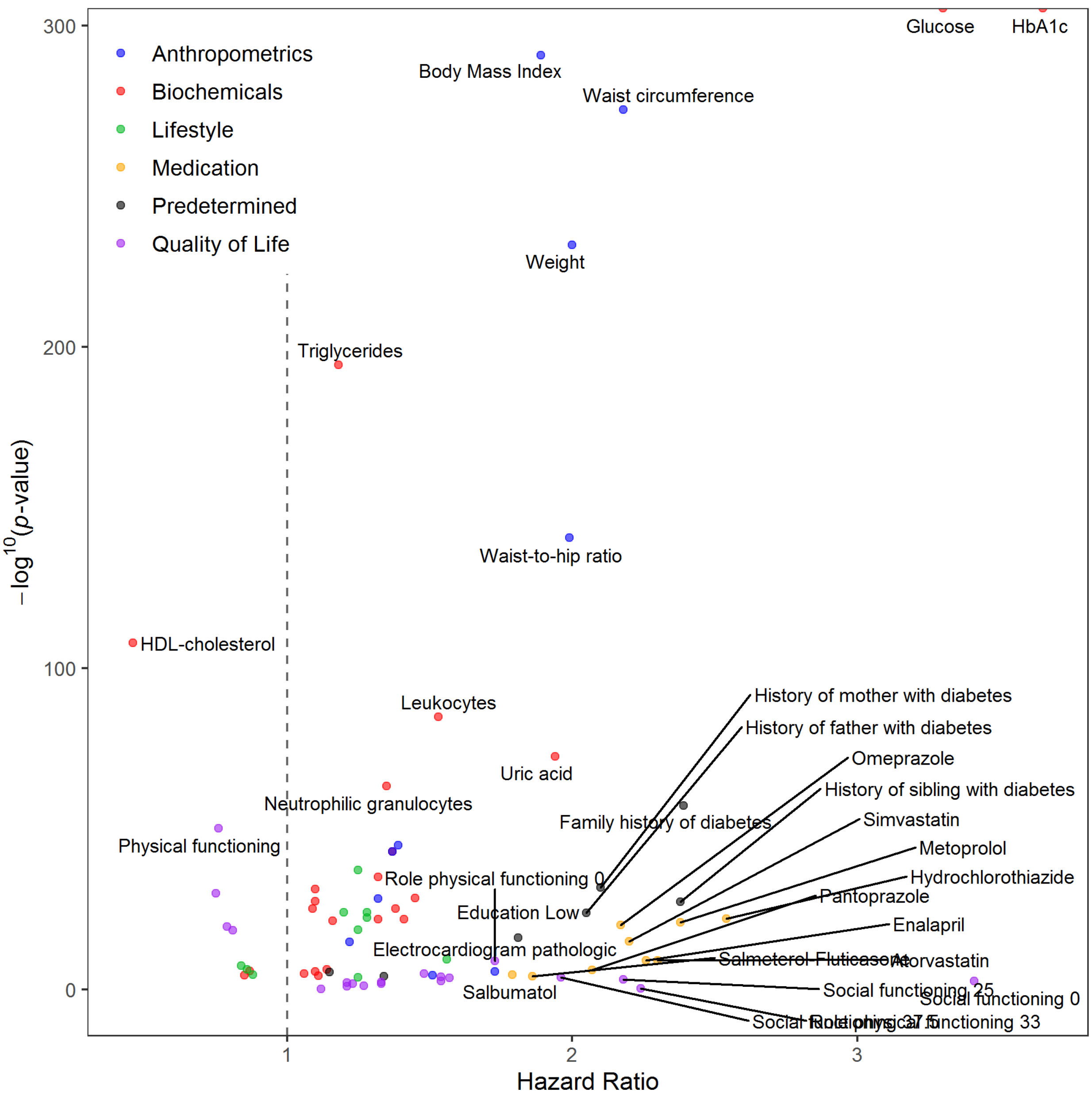

Hazard ratios were calculated using cox-regression and were adjusted for age and sex. All hazard ratios with a larger effect size were annotated.

ESM Figure 3b. Volcano plots of replicated risk variables; Annotated enlargement of **figure 3a**.

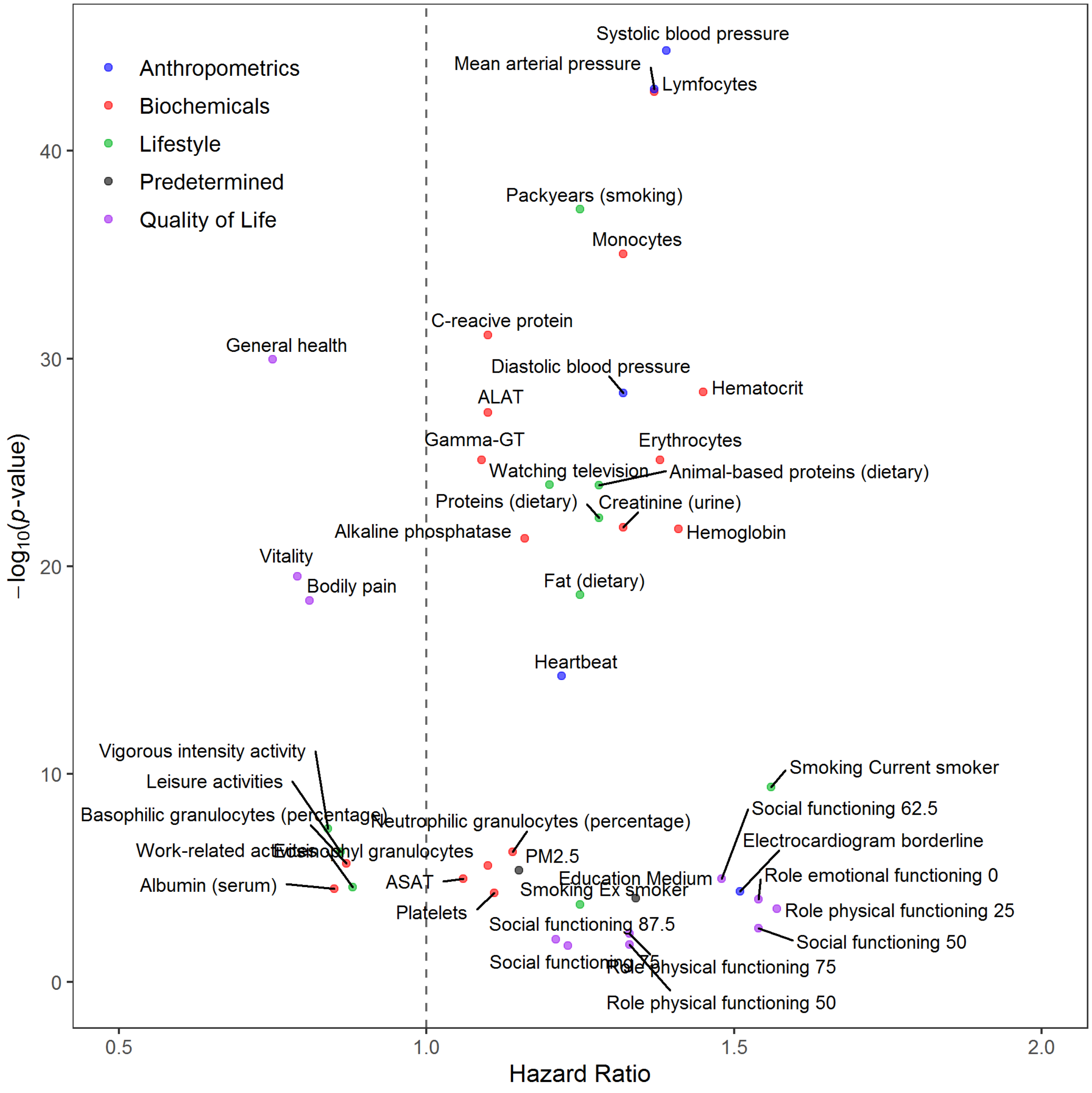

Hazard ratios were calculated using cox-regression and were adjusted for age and sex.

ESM figure 4. The impact of Impaired Fasting Glucose on the hazard ratios of replicated risk variables.

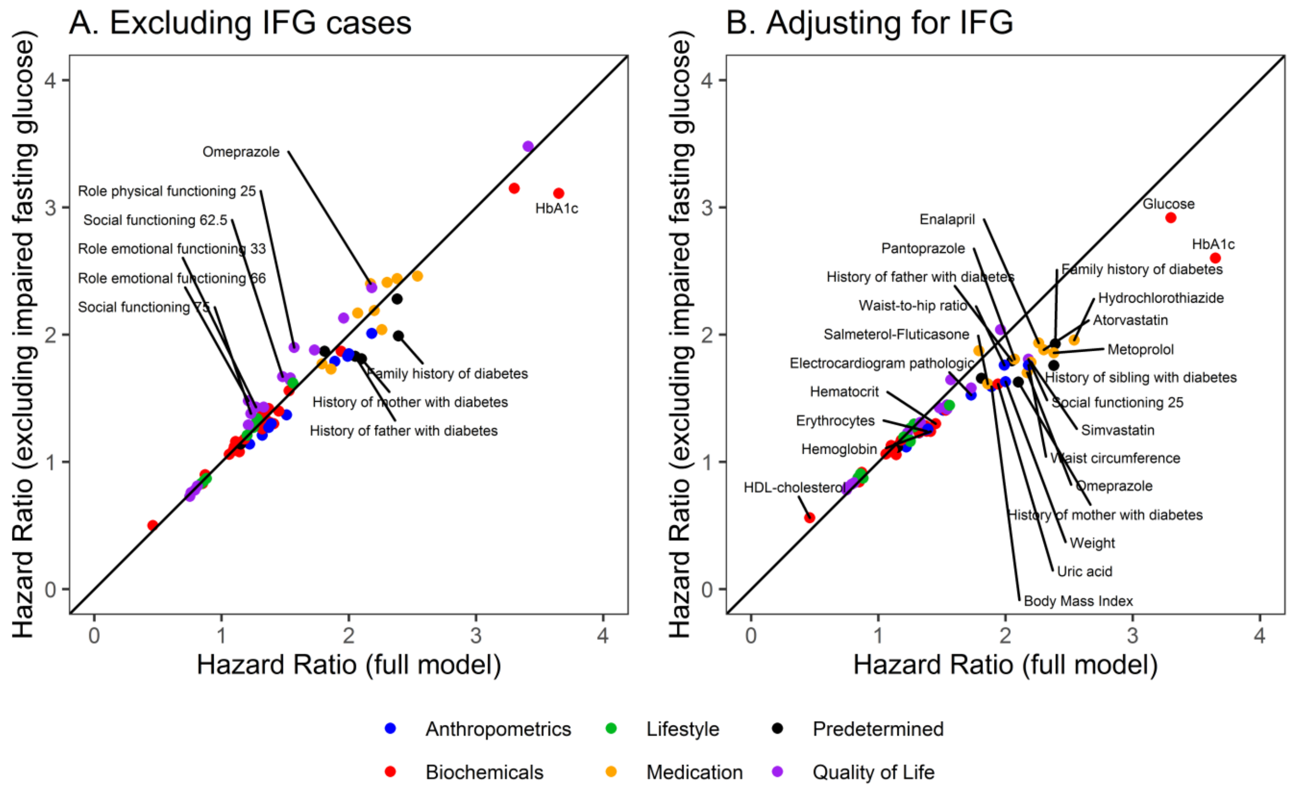

A. We calculated hazard ratios using the full population, which are presented along the x-axis. We also calculated hazard ratios after excluding all with impaired fasting glucose (IFG, defined as fasting glucose >6.0 mmol/L). These are presented along the y-axis. Each dot represents one variable. When a variable is situated above the diagonal axis, the hazard ratio has increased after the exclusion of IFG individuals. When a variable is situated below the diagonal axis, the hazard ratio has decreased after the exclusion of IFG individuals. Differences in hazard ratios greater than 10% are annotated.

B. We calculated hazard ratios using the full population, which are presented along the x-axis. We also calculated hazard ratios while additionally adjusting for IFG (yes/no). Each dot represents one variable. When a variable is situated below the diagonal axis, the hazard ratio has decreased after the additional adjustment of IFG. Differences in hazard ratios greater than 10% are annotated.

ESM figure 5a. Correlation plot of validated risk factors for the development of type 2 diabetes for the full study population.

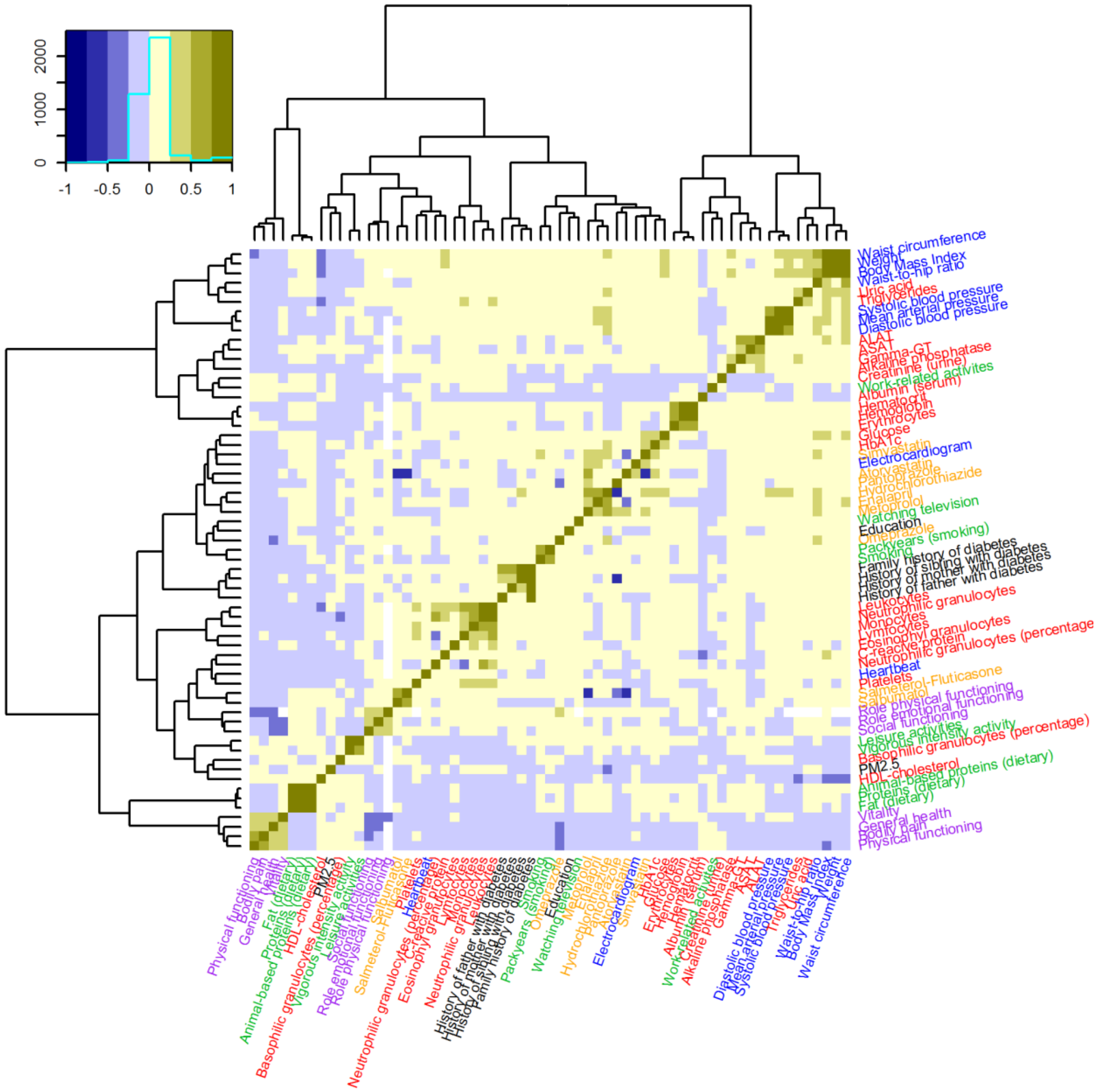

Heterogeneous correlation matrix, consisting of Pearson product-moment correlations, polyserial correlations, and polychoric correlations and arranged using a hierarchical clustering algorithm based on the full dataset. Analyses were performed separate for males (upper triangle) and females (lower triangle). Colours indicate respective groups (blue: anthropometrics; red: biochemicals; green: lifestyle; orange: medication; purple: quality of life; black: predetermined)

ESM figure 5b. Correlation plot of validated risk factors for the development of Type 2 Diabetes for study population of first age tertile (18-39 year).

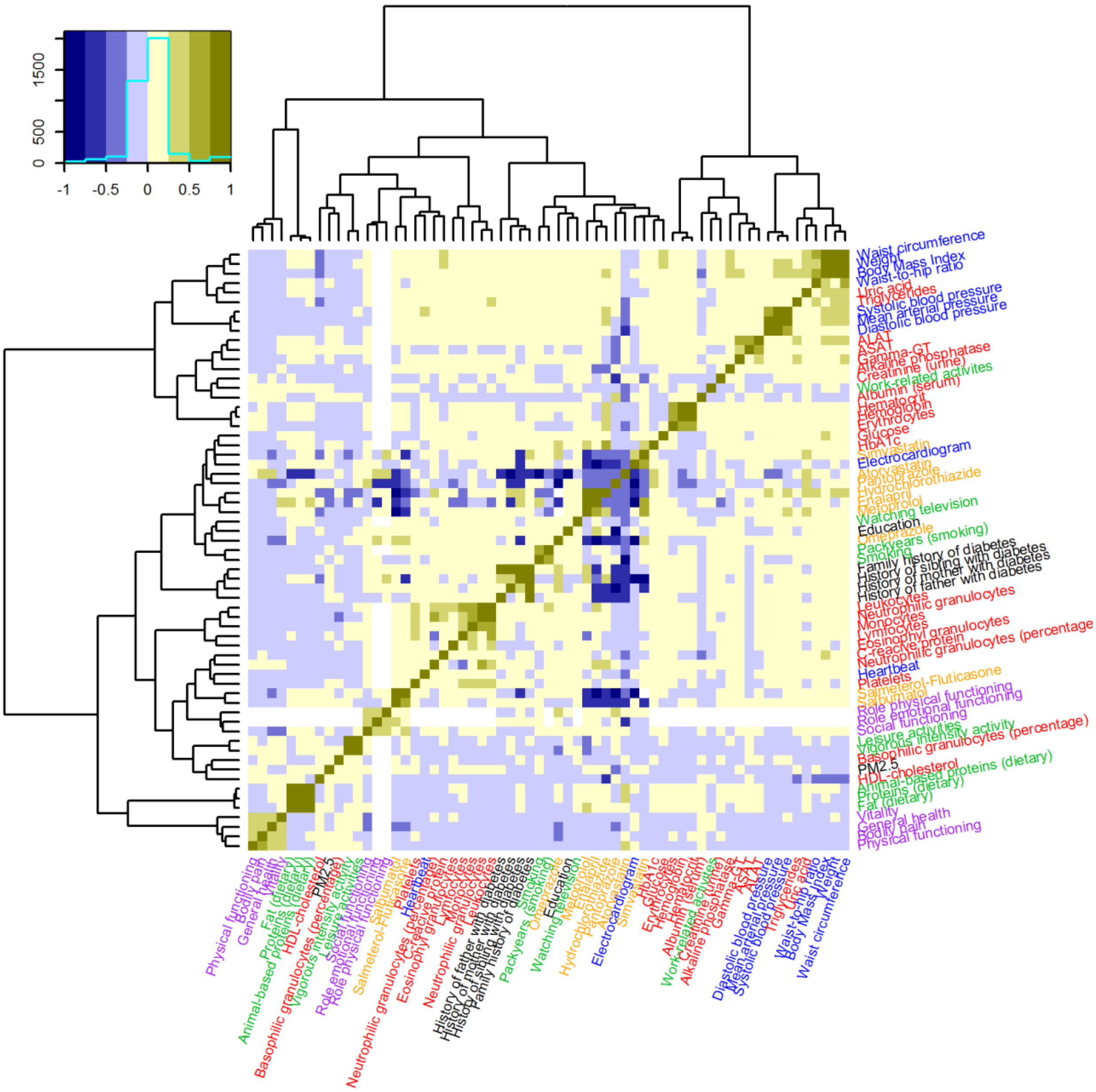

Heterogeneous correlation matrix, consisting of Pearson product-moment correlations, polyserial correlations, and polychoric correlations and arranged using a hierarchical clustering algorithm based on the full dataset. Analyses were performed separate for males (upper triangle) and females (lower triangle). Colours indicate respective groups (blue: anthropometrics; red: biochemicals; green: lifestyle; orange: medication; purple: quality of life; black: predetermined)

ESM figure 5c. Correlation plot of validated risk factors for the development of Type 2 Diabetes for study population of second age tertile (40-48 year).

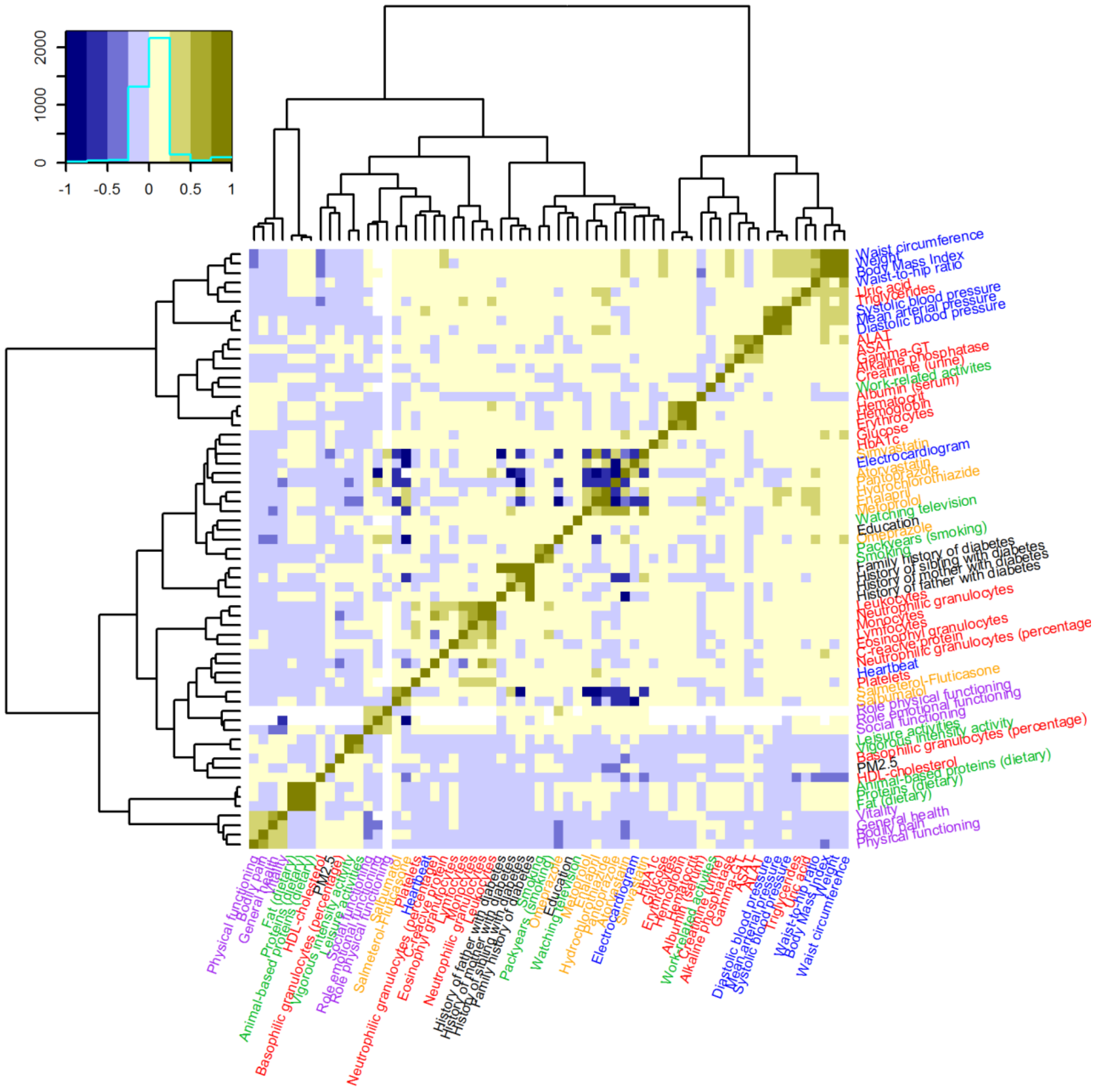

Heterogeneous correlation matrix, consisting of Pearson product-moment correlations, polyserial correlations, and polychoric correlations and arranged using a hierarchical clustering algorithm based on the full dataset. Analyses were performed separate for males (upper triangle) and females (lower triangle). Colours indicate respective groups (blue: anthropometrics; red: biochemicals; green: lifestyle; orange: medication; purple: quality of life; black: predetermined)

ESM figure 5d. Correlation plot of validated risk factors for the development of Type 2 Diabetes for study population of third age tertile (49-91 year).

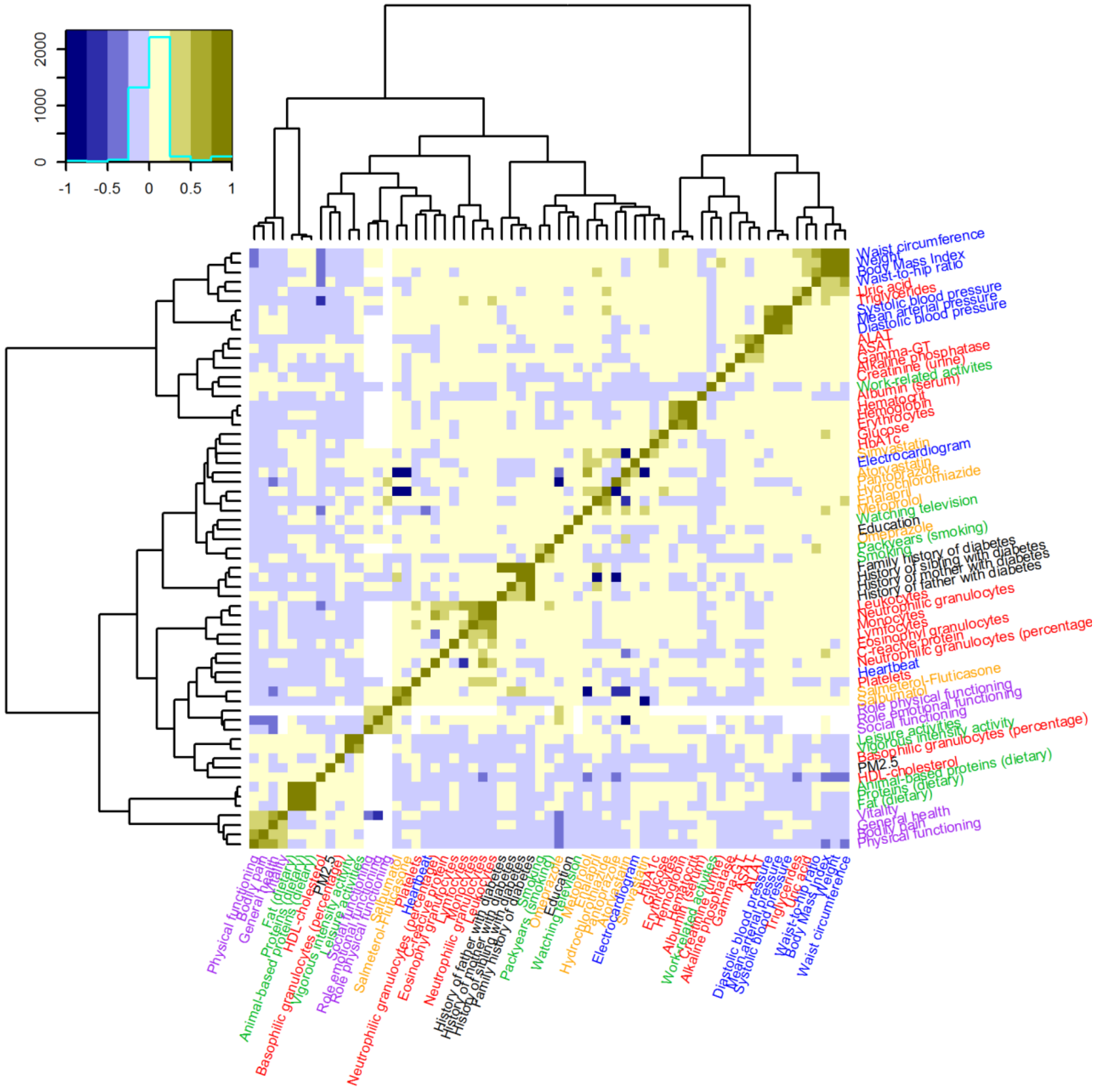

Heterogeneous correlation matrix, consisting of Pearson product-moment correlations, polyserial correlations, and polychoric correlations and arranged using a hierarchical clustering algorithm based on the full dataset. Analyses were performed separate for males (upper triangle) and females (lower triangle). Colours indicate respective groups (blue: anthropometrics; red: biochemicals; green: lifestyle; orange: medication; purple: quality of life; black: predetermined)

ESM figure 6. Trajectories of risk variables included in clinical prediction models.

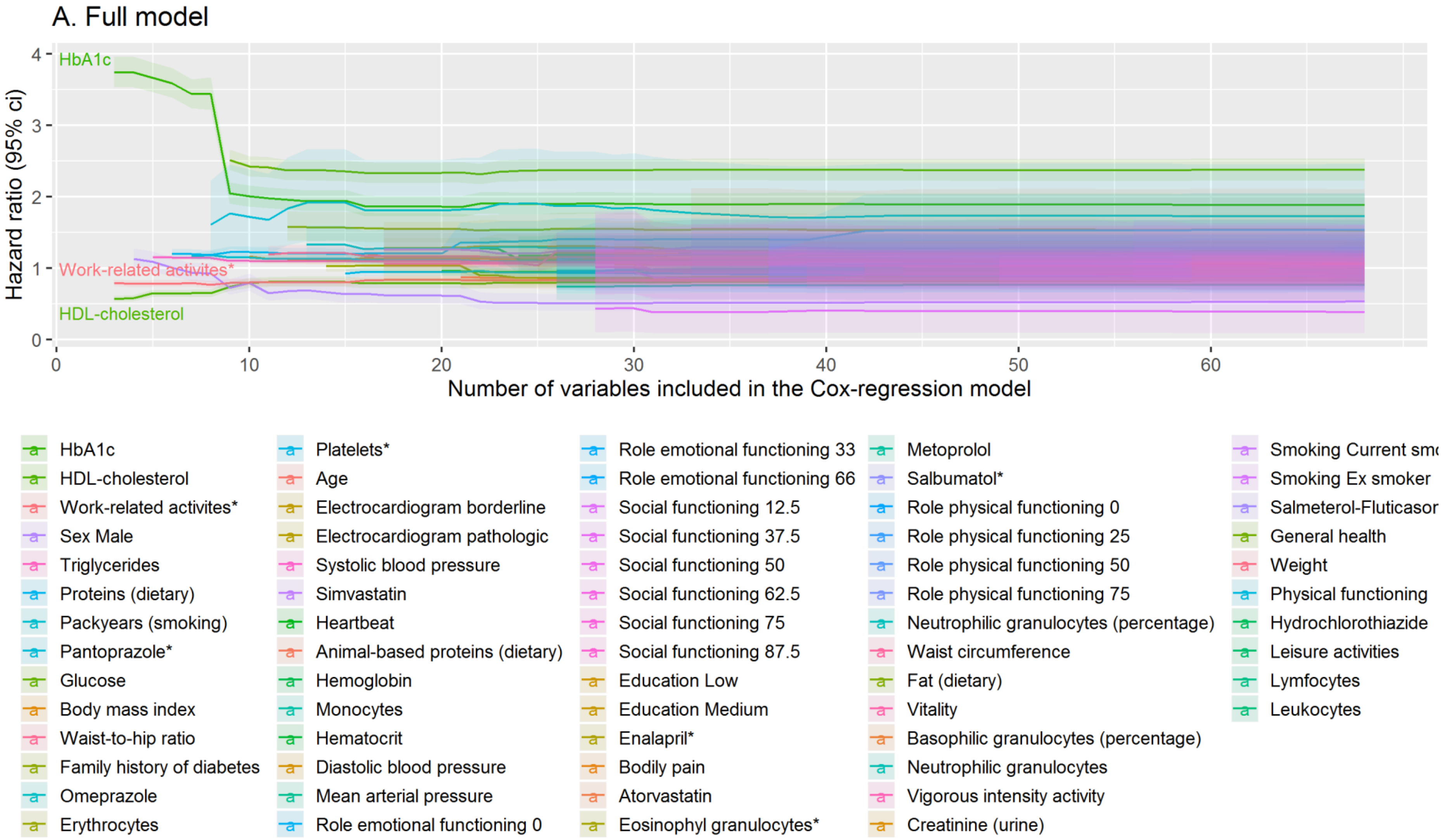

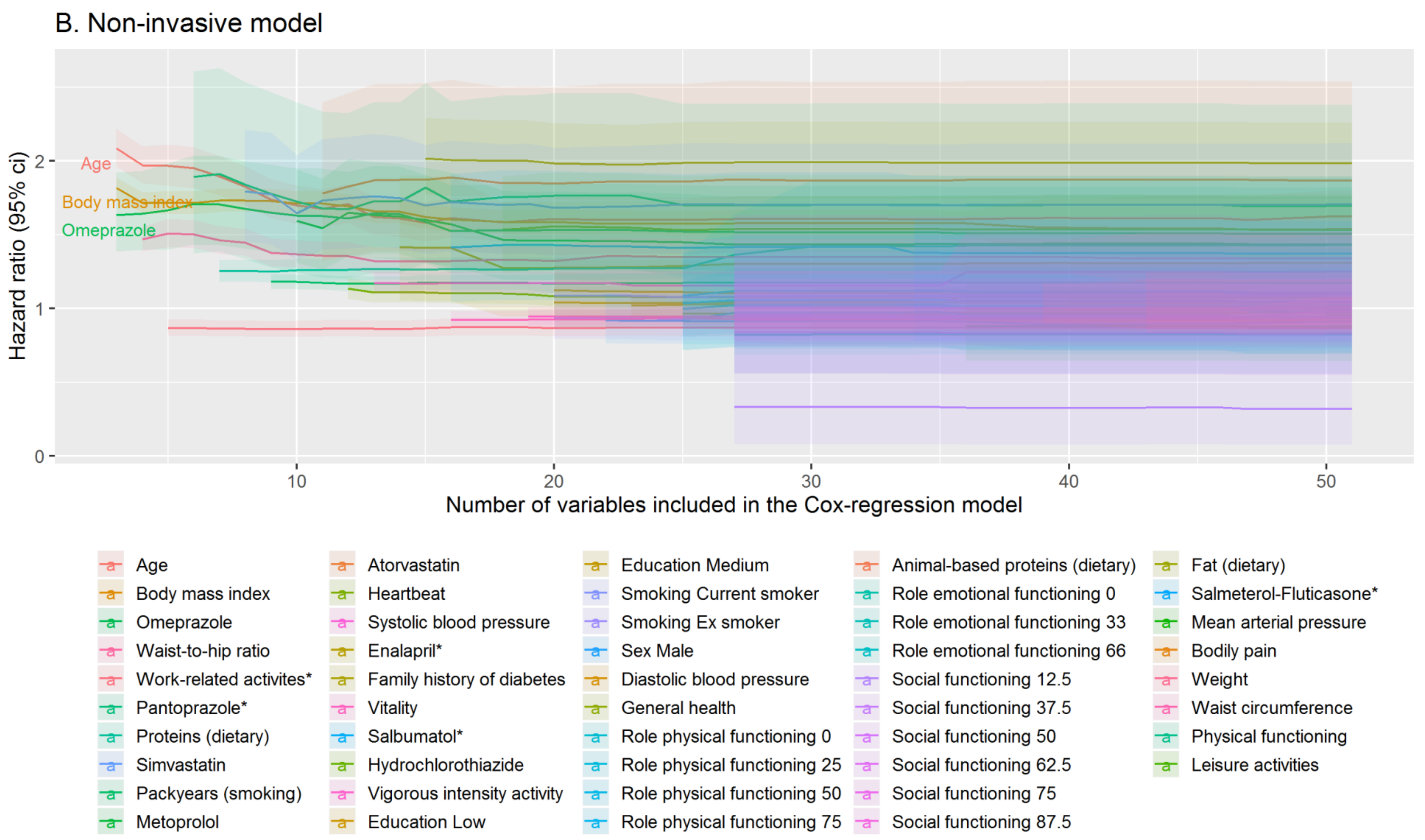
B. Non-invasive model
Hazard ratio (95% ci)
Number of variables included in the Cox-regression model
Age
Body mass index
Omeprazole
0
1
2
10
20
30
40
50
Age
Body mass index
Omeprazole
Waist-to-hip ratio
Work-related activites*
Pantoprazole*
Proteins (dietary)
Simvastatin
Packyears (smoking)
Metoprolol
Atorvastatin
Heartbeat
Systolic blood pressure
Enalapril*
Family history of diabetes
Vitality
Salbumatol*
Hydrochlorothiazide
Vigorous intensity activity
Education Low
Education Medium
Smoking Current smoker
Smoking Ex smoker
Sex Male
Diastolic blood pressure
General health
Role physical functioning 0
Role physical functioning 25
Role physical functioning 50
Role physical functioning 75
Animal-based proteins (dietary)
Role emotional functioning 0
Role emotional functioning 33
Role emotional functioning 66
Social functioning 12.5
Social functioning 37.5
Social functioning 50
Social functioning 62.5
Social functioning 75
Social functioning 87.5
Fat (dietary)
Salmeterol-Fluticasone*
Mean arterial pressure
Bodily pain
Weight
Waist circumference
Physical functioning
Leisure activities

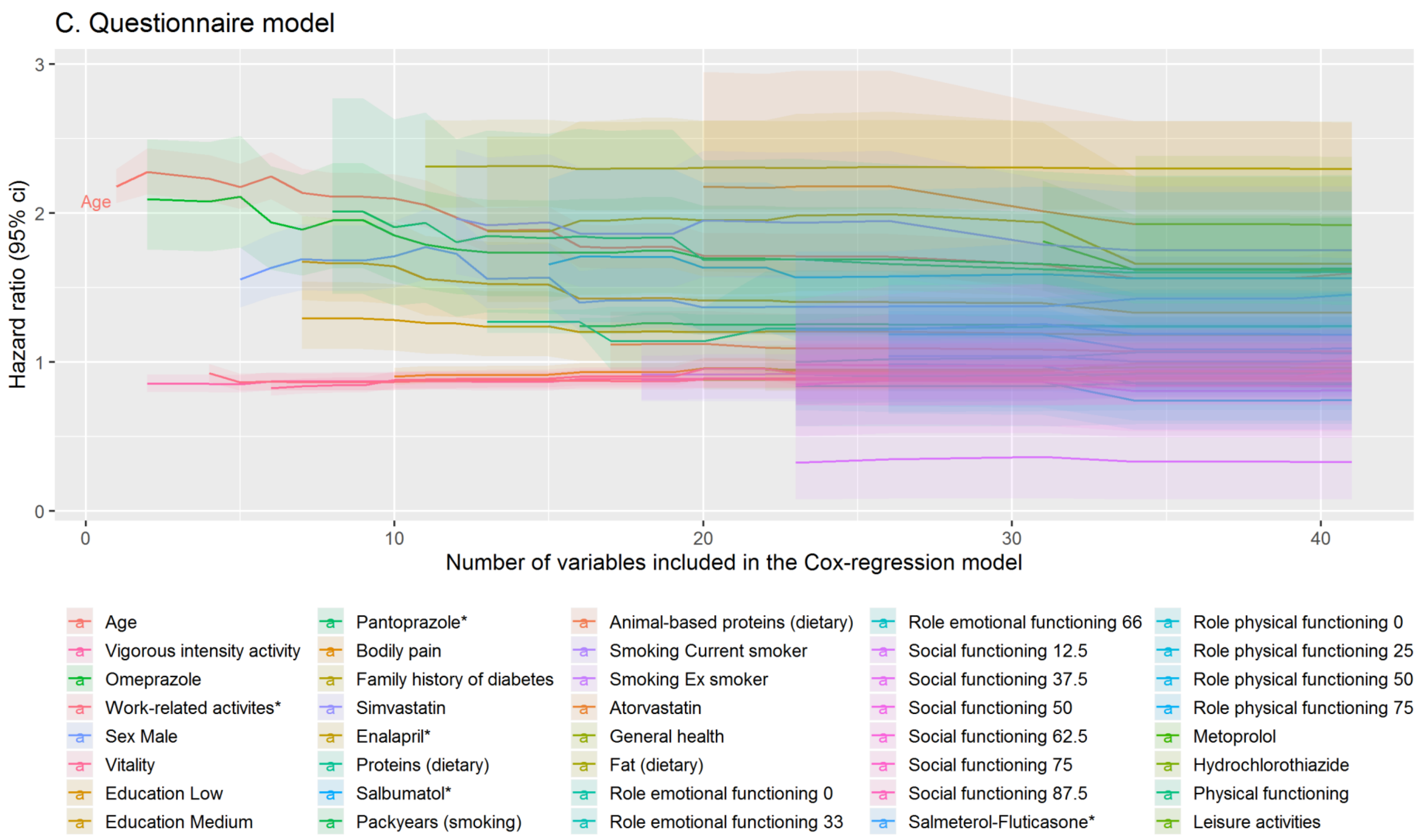

C. Questionnaire model
Hazard ratio (95% ci)
Number of variables included in the Cox-regression model
Age
0
1
2
3
0
10
20
30
40
Age
Vigorous intensity activity
Omeprazole
Work-related activites*
Sex Male
Vitality
Education Low
Education Medium
Pantoprazole*
Bodily pain
Family history of diabetes
Simvastatin
Enalapril*
Proteins (dietary)
Salbumatol*
Packyears (smoking)
Animal-based proteins (dietary)
Smoking Current smoker
Smoking Ex smoker
Atorvastatin
General health
Fat (dietary)
Role emotional functioning 0
Role emotional functioning 33
Role emotional functioning 66
Social functioning 12.5
Social functioning 37.5
Social functioning 50
Social functioning 62.5
Social functioning 75
Social functioning 87.5
Salmeterol-Fluticasone*
Role physical functioning 0
Role physical functioning 25
Role physical functioning 50
Role physical functioning 75
Metoprolol
Hydrochlorothiazide
Physical functioning
Leisure activities

D. Full model (excluding glucose, HbA1c)

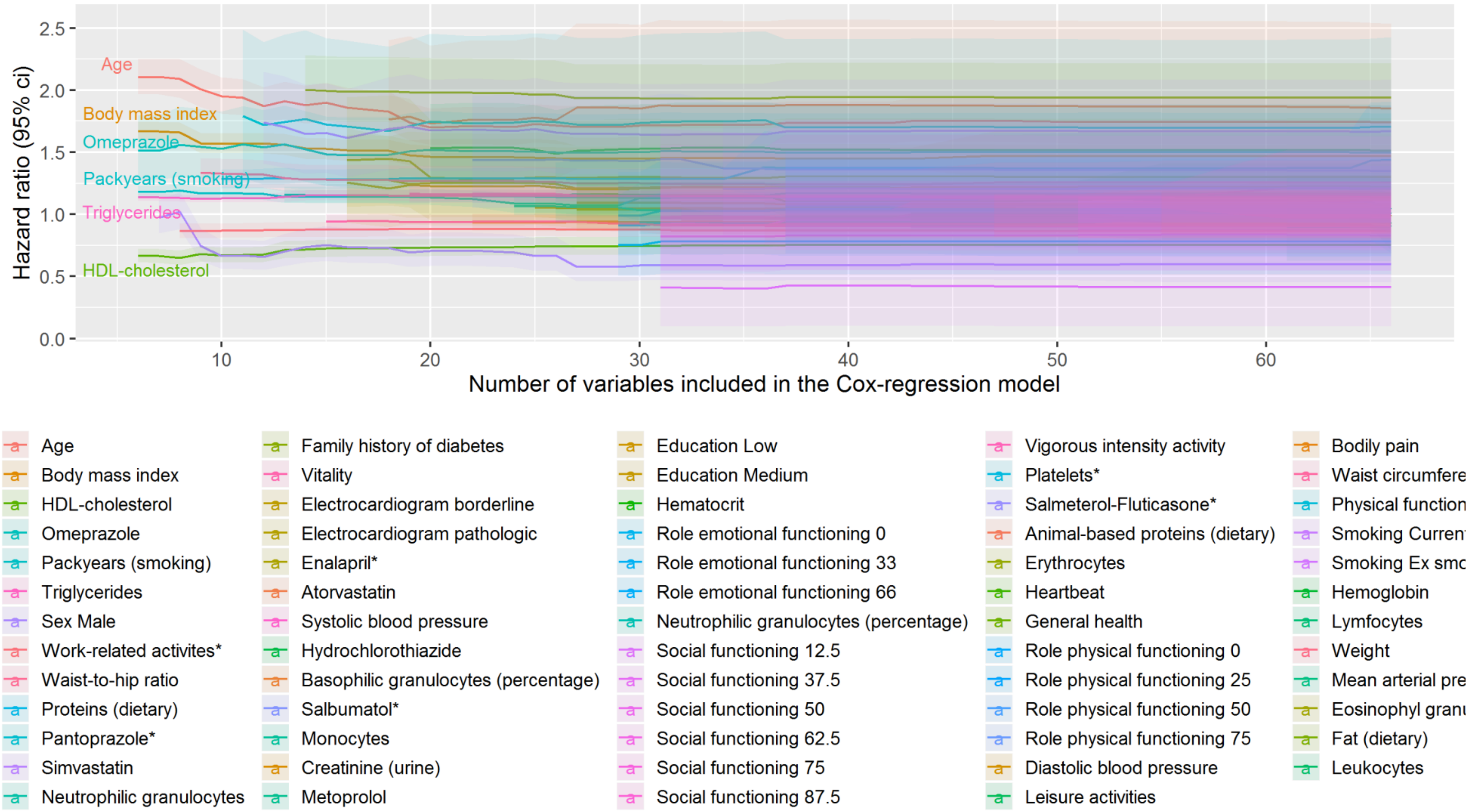

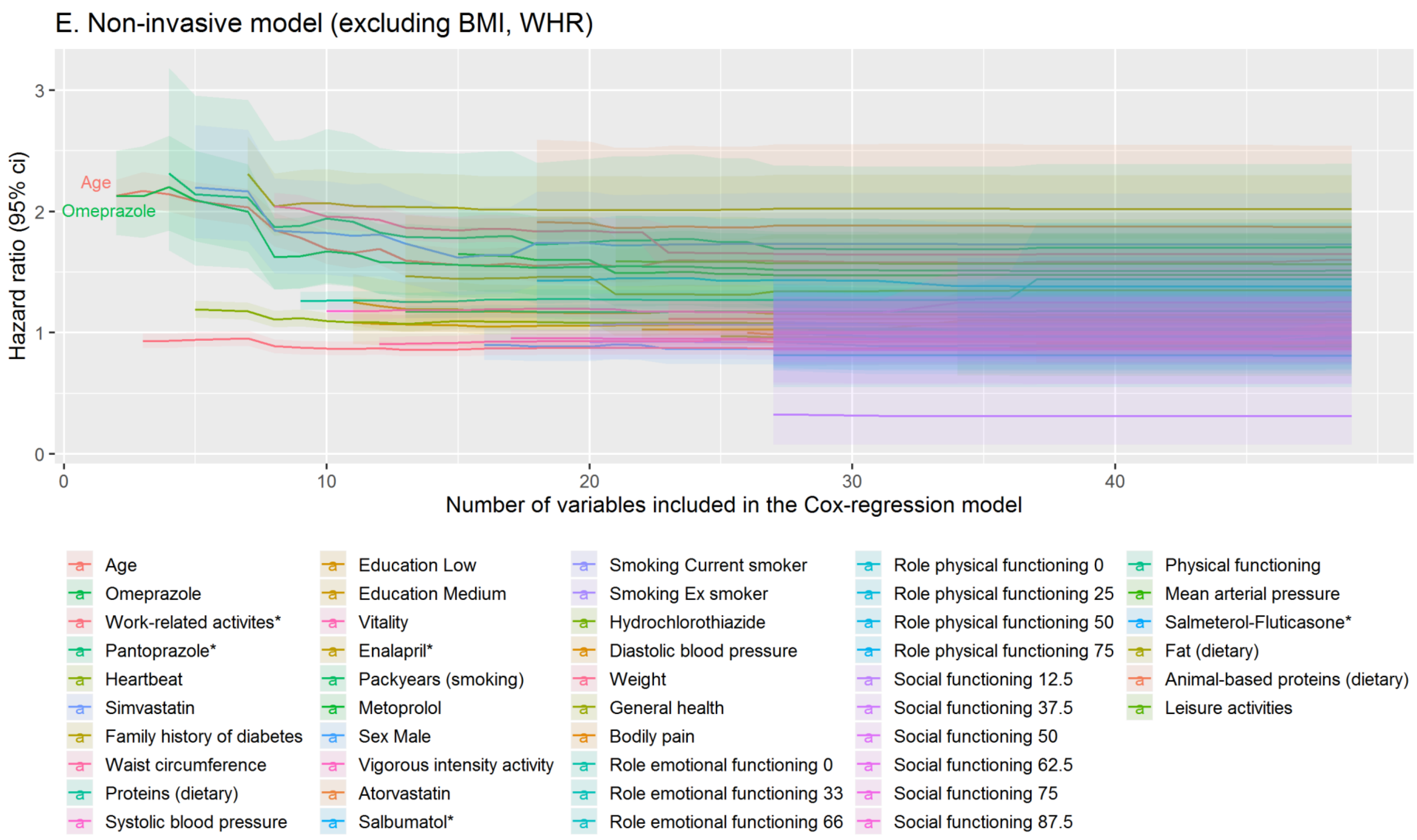
E. Non-invasive model (excluding BMI, WHR)
Hazard ratio (95% ci)
Number of variables included in the Cox-regression model
0
1
2
3
0
10
20
30
40
Age
Omeprazole
Age
Omeprazole
Work-related activites*
Pantoprazole*
Heartbeat
Simvastatin
Family history of diabetes
Waist circumference
Proteins (dietary)
Systolic blood pressure
Education Low
Education Medium
Vitality
Enalapril*
Packyears (smoking)
Metoprolol
Sex Male
Vigorous intensity activity
Atorvastatin
Salbumatol*
Smoking Current smoker
Smoking Ex smoker
Hydrochlorothiazide
Diastolic blood pressure
Weight
General health
Bodily pain
Role emotional functioning 0
Role emotional functioning 33
Role emotional functioning 66
Role physical functioning 0
Role physical functioning 25
Role physical functioning 50
Role physical functioning 75
Social functioning 12.5
Social functioning 37.5
Social functioning 50
Social functioning 62.5
Social functioning 75
Social functioning 87.5
Physical functioning
Mean arterial pressure
Salmeterol-Fluticasone*
Fat (dietary)
Animal-based proteins (dietary)
Leisure activities

Each line represents the hazard ratio and 95% confidence interval of a risk variable in: A. a full model (i.e. including all variables), B. a non-invasive model, C. a questionnaire model, D. a full model (excluding glucose, HbA1c), and E. a non-invasive model (excluding body-mass index and waist-to-hip ratio). Risk variables were added to the model based on their robustness in the bootstrapped lasso-regression analysis. A change in a line represents the modification in hazard ratio of the respective variable when a new risk variable is added to the model. Variables first added to the model were annotated. The legend depicts the variables in the order they were added to the model.
